# Identification of RimR2 as a positive pathway-specific regulator of rimocidin biosynthesis in *Streptomyces rimosus* M527

**DOI:** 10.1186/s12934-023-02039-9

**Published:** 2023-02-21

**Authors:** Huijie Li, Yefeng Hu, Yongyong Zhang, Zheng Ma, Andreas Bechthold, Xiaoping Yu

**Affiliations:** 1 Zhejiang Provincial Key Laboratory of Biometrology and Inspection & Quarantine, College of Life Sciences, China Jiliang University, Xueyuan Street, Xiasha Higher Education District, Hangzhou, 310018 Zhejiang People’s Republic of China; 2 Institute for Pharmaceutical Sciences, Pharmaceutical Biology and Biotechnology, University of Freiburg, 79104 Freiburg, Germany

**Keywords:** Rimocidin, RimR2, LAL regulator, *Streptomyces rimosus*

## Abstract

**Background:**

*Streoptomyces rimosus* M527 is a producer of the polyene macrolide rimocidin which shows activity against various plant pathogenic fungi. Notably, the regulatory mechanisms underlying rimocidin biosynthesis are yet to be elucidated.

**Results:**

In this study, using domain structure and amino acid alignment and phylogenetic tree construction, *rimR*2, which located in the rimocidin biosynthetic gene cluster, was first found and identified as a larger ATP-binding regulators of the LuxR family (LAL) subfamily regulator. The *rimR*2 deletion and complementation assays were conducted to explore its role. Mutant M527-ΔrimR2 lost its ability to produce rimocidin. Complementation of M527-ΔrimR2 restored rimocidin production. The five recombinant strains, M527-ER, M527-KR, M527-21R, M527-57R, and M527-NR, were constructed by overexpressing *rimR*2 gene using the promoters p*erm*E^*^, *kasO*p^*^, SPL21, SPL57, and its native promoter, respectively, to improve rimocidin production. M527-KR, M527-NR, and M527-ER exhibited 81.8%, 68.1%, and 54.5% more rimocidin production, respectively, than the wild-type (WT) strain, while recombinant strains M527-21R and M527-57R exhibited no obvious differences in rimocidin production compared with the WT strain. RT-PCR assays revealed that the transcriptional levels of the *rim* genes were consistent with the changes in rimocidin production in the recombinant strains. Using electrophoretic mobility shift assays, we confirmed that RimR2 can bind to the promoter regions of *rimA* and *rimC.*

**Conclusion:**

A LAL regulator RimR2 was identified as a positive specific-pathway regulator of rimocidin biosynthesis in M527. RimR2 regulates the rimocidin biosynthesis by influencing the transcriptional levels of *rim* genes and binding to the promoter regions of *rimA* and *rimC*.

**Supplementary Information:**

The online version contains supplementary material available at 10.1186/s12934-023-02039-9.

## Background

Polyketides, a large group of secondary metabolites synthesized by polyketide synthases (PKSs), exhibit various bioactivities, including antifungal (rimocidin), antibacterial (penicillin), antitumor (daunorubicin) properties [1–3]. They are naturally present in bacteria, fungi, plants, protists, insects, mollusks, and sponges. *Streptomyces*, a genus of Gram-positive bacteria with three types of PKSs (types I, II, and III), is best known for producing polyketides [4–6]. Polyene macrolide antibiotics (PEM) are primarily synthesized by type I PKS and are very effective antifungal drugs [7–9], They include natamycin [10], nystatin [11], amphotericin [12], and rimocidin, all of which contain a macrolide ring with a sugar moiety. The primary target of PEM is the fungal cell membrane, which interacts with PEM via the ergosterol-forming channels present on it, causing loss of ions, imbalance of electrochemical gradients, and cell death [13]. For example, rimocidin, which exhibits excellent antagonistic activity against various plant pathogenic fungi, especially *Fusarium oxysporum* f. sp. *cucumerinum* [14], is a promising agricultural antibiotic as it is difficult to develop drug resistance.

However, polyketide biosynthesis in *Streptomyces* causes bottlenecks, leading to low production levels and long fermentation periods [15]. Secondary metabolite biosynthesis regulation in *Streptomyces* is a complex process involving multiple levels [16–18], including biosynthetic pathway regulation, wherein pathway-specific regulatory genes located in their respective biosynthetic gene clusters regulate biosynthetic gene expression, consequently affecting secondary metabolite production [19–21].

To date, the different types of regulators involved in polyene macrolide biosynthesis have been categorized as follows: (1) *Streptomyces* antibiotic regulatory protein (SARP) family regulator, such as ActII-orf4, which regulates actinorhodin biosynthesis, and CcaR, which regulates clavulanic biosynthesis. These regulators are characterized by the presence of OmpR -like DNA-binding domains [22]. (2) PAS-LuxR regulators, which combine an N-terminal PAS sensory domain with a C-terminal helix-turn-helix (HTH) motif of the LuxR type [23, 24]. The PAS domain is considered capable of sensing various environmental factors, such as light, oxygen and redox potentials. Examples of PAS-LuxR regulators include PimM, which regulates pimaricin biosynthesis in *Streptomyces natalensis* [25]. AmphRIV, which regulates amphotericin biosynthesis in *Streptomyces nodosus* [26], and NysRIV, which regulates nystatin biosynthesis in *Streptomyces noursei* [27]. (3) Larger ATP-binding regulators of the LuxR (LAL) family regulators, which are characterized by an unusually large number of amino acids (~ 900) [28] having an ATP-binding motif near the N-terminal end and Walker A, Walker B, and a HTH domain at the C-terminus. Numerous regulators belonging to the LAL family have been identified, such as TtmRIII, which regulates tetramycin biosynthesis in *Streptomyces ahygroscopicus* [29], and NysRI, NysRII, and NysRIII, which regulates nystatin biosynthesis in *S. noursei* ATCC 11,455 [30]. (4) SARP-LAL regulators, which combine an N-terminal DNA-binding domain corresponding to the SARP family with a C-terminal half that is similar to the LAL regulators. Examples of SARP-LAL regulators include PimR, which regulates pimaricin biosynthesis in *S. natalensis* [31], and PteR, which regulates filipin biosynthesis in *Streptmyces avermilitis* [32]. Recent studies have stated that the elucidation of regulatory mechanisms at the molecular level forms a foundation for improving secondary metabolite production [33–35].

*Streptomyces rimosus* M527, a rimocidin producer, was originally isolated by Lu et al. [14] and deposited in the China Center for Type Culture Collection (M2013270). However, the low production of rimocidin in this strain precludes its application in large-scale industrial production. Recently, some strategies, including ribosome engineering [36], fermentation condition optimization, and elicitors addition [37], were applied to improve rimocidin production in *S. rimosus* M527. However, to the best of our knowledge, no pathway-specific rimocidin biosynthesis regulators are currently known.

**Fig. 1:**
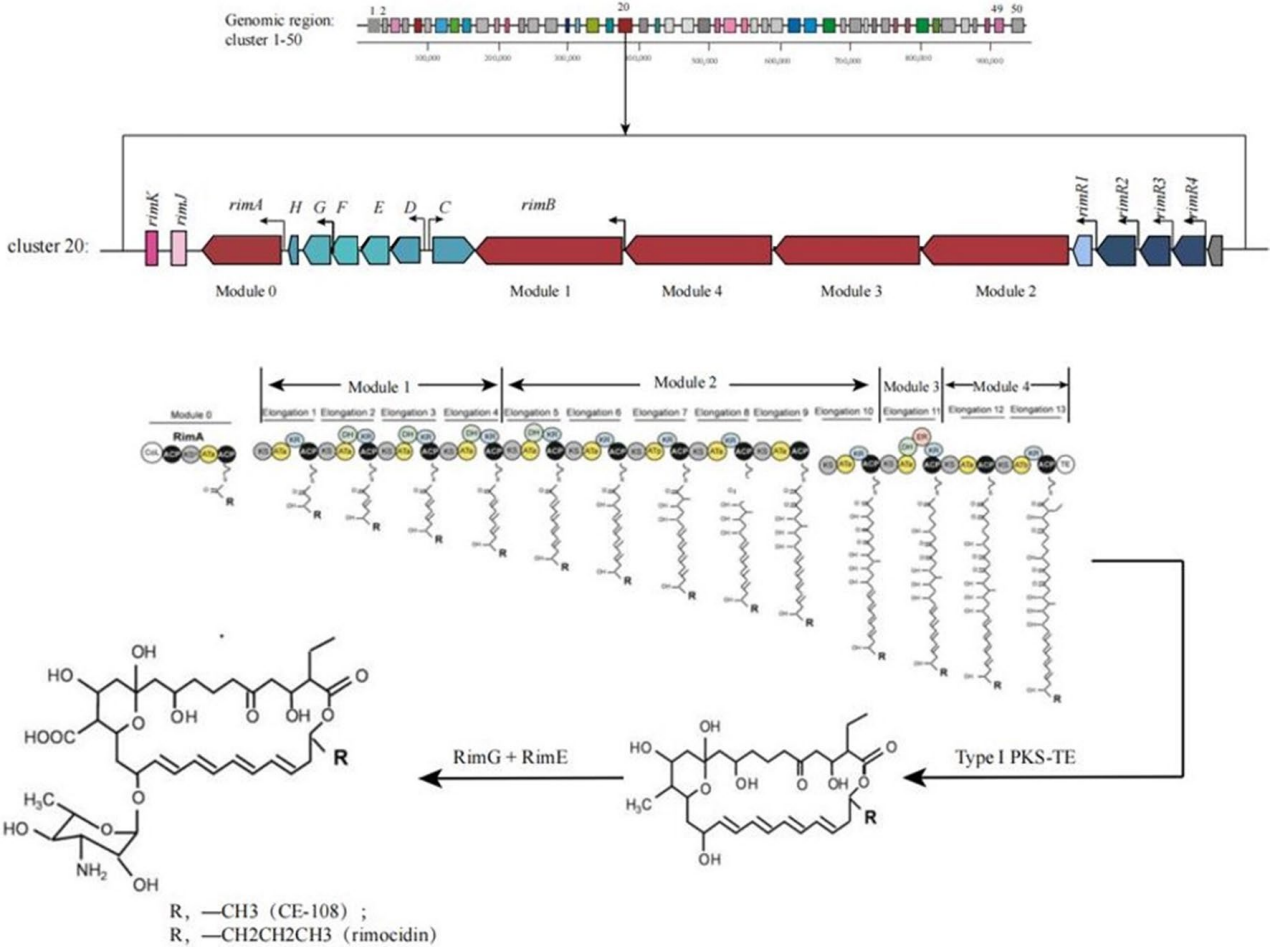
Gene organization of *rim* gene cluster in the genome of *Streptomyces rimosus* M527 and rimocidin biosynthetic pathway. Module 0, Module 1, Module 2, Module 3, Module 4, type I polyketide synthase; *rimK*, acetyltransferase; *rimJ*, crotony-CoA reductase; *rimH*, ferredoxin; *rimG*, cytochrome P450 monooxygenase; *rimF*, aminotransferase; *rimE*, glycosyl transferase; *rimD*, cholesterol oxidase; *rimC*, tyrosine phosphatase; *rimR*l, PAS-LuxR family transcriptional regulator; *rimR*2, *rimR*3, *rimR*4, LAL family transcriptional regulator. The arrows in gene cluster represent putative promoters. Proposed model for rimocidin and CE-108 biosynthesis in *S. diastaticus* var. 108 [38]

Although the rimocidin biosynthetic pathway in *Streptomyces diastaticus* var. 108 has been predicted, and its biosynthetic gene cluster has been published (GenBank Accession No. AY442225) [38], no pathway-specific regulatory gene has been discovered. Recently, the whole genome of *S. rimosus* M527 was sequenced (GenBank Accession No: NZ_SADA00000000.1), a biosynthetic gene cluster responsible for rimocidin production (GenBank Accession No. MK300953) (Fig. 1) was detected. This gene cluster contains four regulatory genes named *rimR*1*-rimR*4. As RimR2 is predicted to be a positive regulator we decided to its function by gene deletion and complementation experiments. We compared rimocidin production, cell growth, and the relative transcriptional levels of structural gene among wide-type (WT), *rimR*2-deleted and *rimR*2-complemented strains. Subsequently, *rimR*2 gene was overexpressed using different promoters (permE^*^, kasOp^*^, SPL21, SPL57 and its native promoter) to improve rimocidin production. Furthermore, the regulatory mechanism of RimR2 was identified using electrophoretic mobility shift assays (EMSA).

## Results

### RimR2 is a LuxR-family transcription regulator and is indispensable to rimocidin biosynthesis

**Fig. 2:**
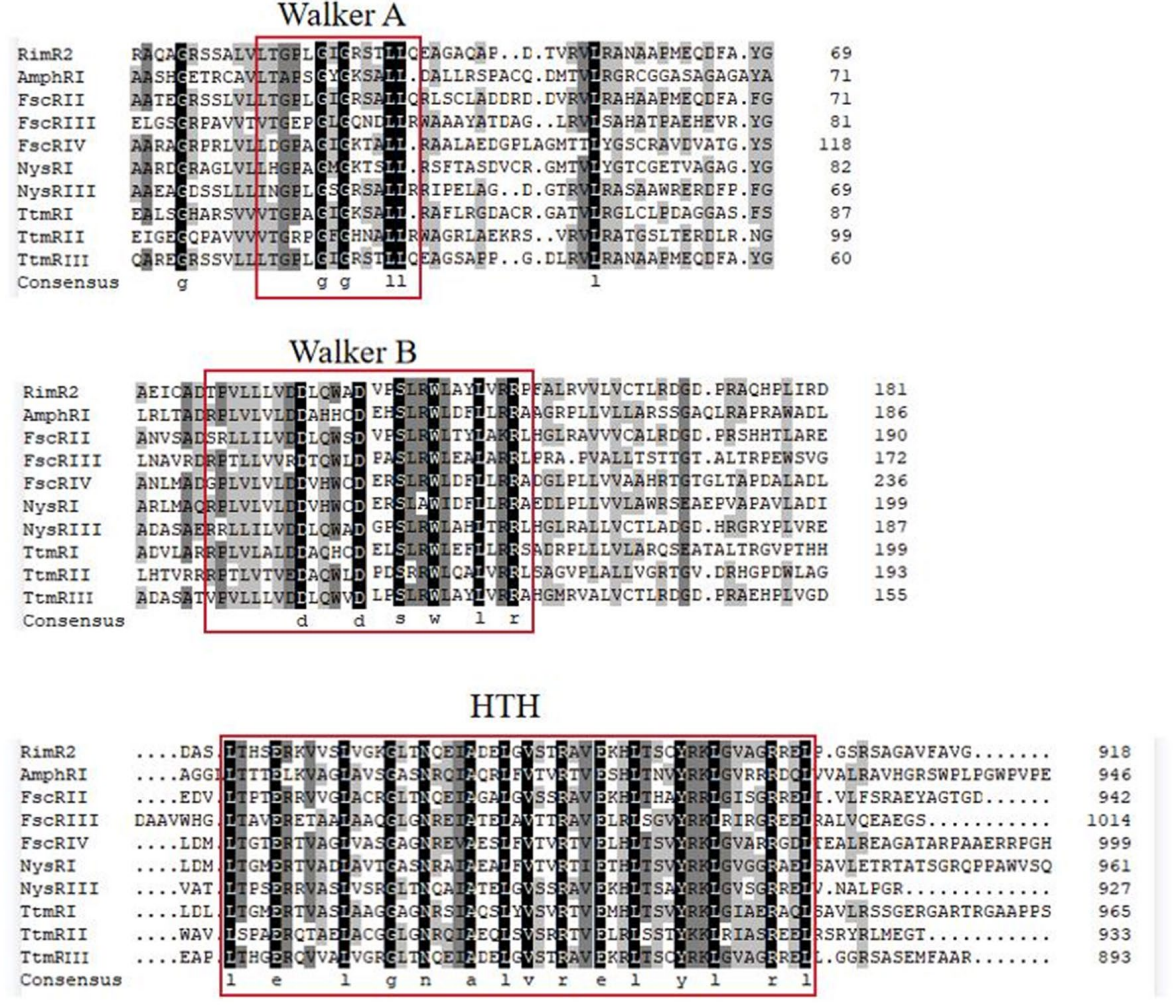
Domain structure and amino acid alignment of RimR2 and related LAL family regulators. Sequence comparisons of the N-terminal Walker A and Walker B domains and C-terminal HTH between RimR2 and well-studied LAL family regulators. AmphRI, a regulator of Amphotericin biosynthesis from *Streptomyces nodosus*; FscRII, FscRIII, FscRIV, regulators of Candicidin biosynthesis from *Streptomyces* sp. FR-008; NysRI, NysRIII, regulators of nystatin biosynthesis from *Streptomyces noursei* ATCC 11455; TtmRI, TtmRII, TtmRIII, regulators of tetramycin biosynthesis in *Streptomyces ahygroscopicus*. The NCBI database accession numbers of the sequences used in this analysis are as follows: AAV37059.1(AmphRI), AAQ82552.1(FscRII), AAQ82553.1(FscRIII), AAQ82554.1 (FscRIV), AAF71778.1(NysRI), AAF71780.1 (NysRIII), AFW98290.1(TtmRI), AFW98288.1(TtmRII), and AFW98289.1 (TtmRIII)

**Fig. 3:**
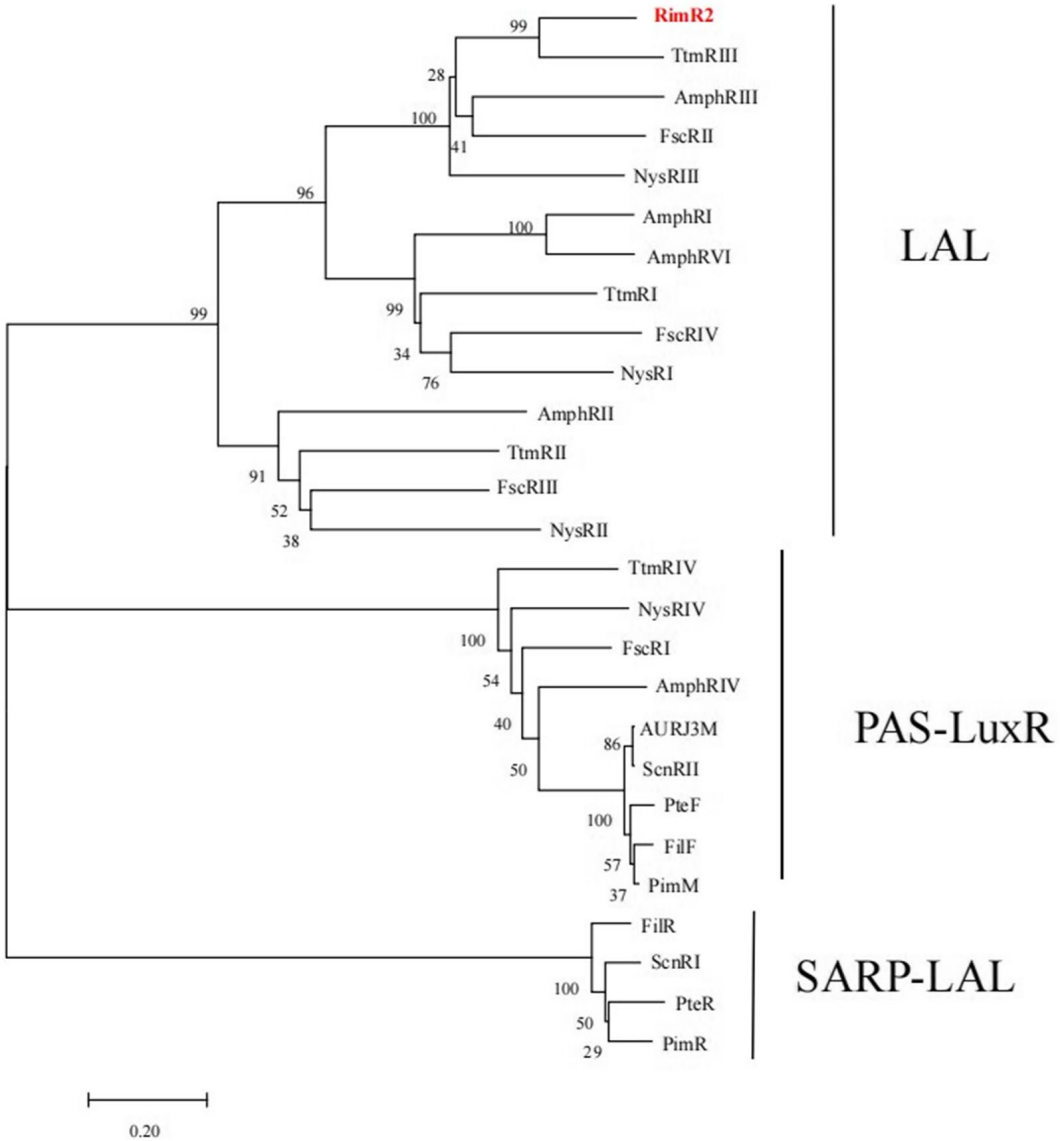
Phylogenetic analysis based on RimR2 of *S. rimosus* M527 and some polyene macrolide biosynthesis regulators from other *Streptomyces* species. Phylogenetic analysis was performed with MEGA 7.0, using the neighbor-joining method in the Jukes-Cantor model. Bootstrap values (> 50%) based on 1000 replicates were shown at the branch nodes. Bar, 0.20 substitutions per nucleotide positions

According to the *S. rimosus* M527 genome sequence, *rimR*2 gene (2757 nucleotides (nt)), located in the rimocidin biosynthesis gene cluster, encodes a protein with a predicted molecular mass of 97.3 kDa consisting of 918 aa. RimR2 protein contains a conserved nucleotide phosphate-binding domain (Walker A and Walker B) and an HTH DNA-binding domain (Fig. 2). It resembles proteins of the LAL family regulator widely distributed in *Streptomyces* species. Among them, RimR2 is most similar to TtmRIII from *S. ahygroscopicus* (AFW98289.1, 70.62% identity) (Fig. 3 and Additional file 1: Table S1). Collectively, the above results suggest that RimR2 belongs to the LAL family of transcriptional regulators.

The *rimR*2-deleted mutant M527-ΔrimR2 was constructed by using the CRISPR/Cas9-CodA (sm) method (Additional file 2: Figure S1). A 2.8-kb band was obtained by polymerase chain reaction (PCR) using the M527 strain as template, whereas no fragment was obtained using mutant M527-ΔrimR2 as template, thereby confirming the successful construction of the *rimR*2 deletion mutant (Additional file 3: Figure S2).

The growth and morphology of WT strain and mutant M527-ΔrimR2 cultured on MS agar media were identical, indicating that *rimR*2 did not significant affect on cell growth. The rimocidin yield was determined from shake-flask fermentation cultures of both strains. High-performance liquid chromatography (HPLC) analysis revealed that the mutant M527-ΔrimR2 could not produce any rimocidin, whereas a distinct rimocidin peak was clearly observed in the WT culture filtrates (Additional file 4: Figure S3).

To confirm that *rimR*2 deletion was solely responsible for this difference, a 3.1-kb DNA fragment containing *rimR*2 and its promoter region was reintroduced into M527-ΔrimR2 using the plasmid pSET152::*rimR*2, yielding the complemented strain M527-ΔrimR2/pSET152::*rimR*2*.* Under standard fermentation conditions, the complemented strain produced rimocidin at a level comparable with that produce by the WT strain (Additional file 4: Figure S3), thus validating that *rimR*2 is essential for rimocidin biosynthesis in *S. rimosus* M527.

**Fig. 4:**
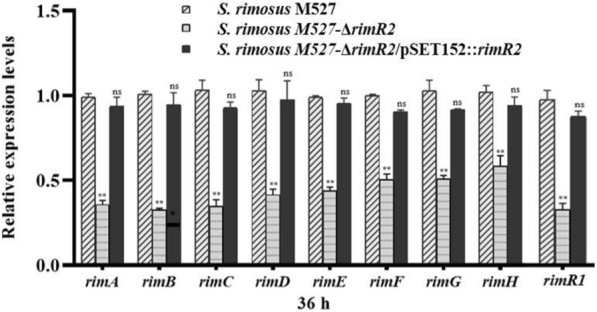
Comparison of the transcriptional levels of *rim* genes involved in rimocidin biosynthetic gene cluster by using qRT-PCR in WT strain *S. rimosus* M527, mutant *S. rimosus* M527-ΔrimR2, complemented strain *S. rimosus* M527-ΔrimR2/pSET152::*rimR*2.^**^indicates highly statistically significant results (*P*-value < 0.01)

Quantitative reverse transcription-PCR (qRT-PCR) was performed to examine the effects of *rimR*2 deletion on the transcriptional levels of rimocidin biosynthetic genes (*rim* genes) located in the gene cluster. The mutant M527-ΔrimR2 exhibited significantly lesser transcriptional levels of all the candidate *rim* genes than the WT strain (Fig. 4), and the transcriptional levels were restored in M527-ΔrimR2 containing *rimR*2 for complementation. Thus, these results suggest that *rimR*2 is a pathway-specific positive regulator of rimocidin biosynthesis in *S. rimosus* M527.

### RimR2 protein binds specifically to the promoter regions of* rimA* and *rimC*

**Fig. 5:**
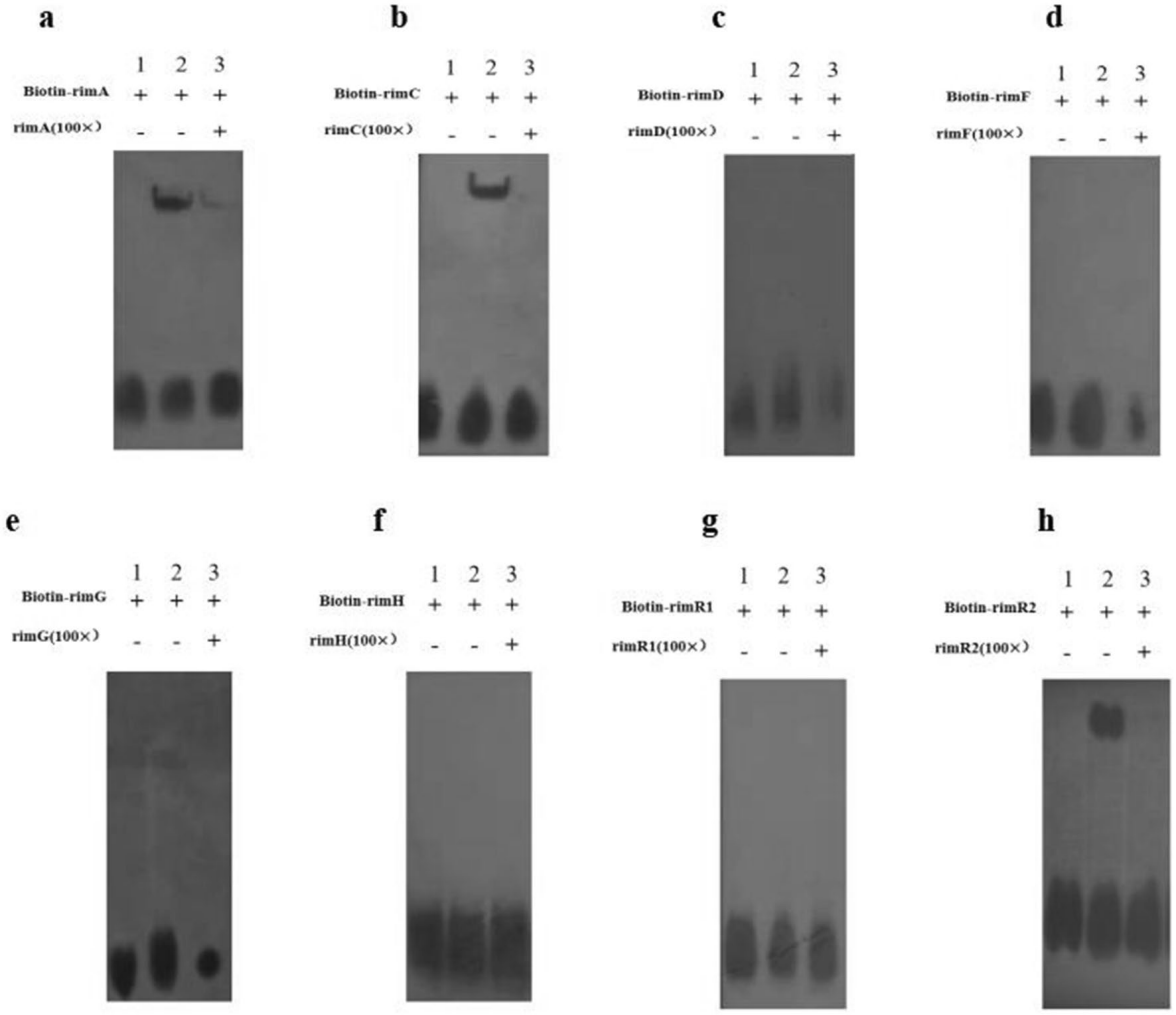
In vitro electrophoretic mobility-shift assay (EMSA) assay of RimR2 binding to the promoter regions of the rimocidin biosynthetic genes *rimA*(**a**), *rimC* (**b**), *rimD* (**c**), *rimF* (**d**), *rimG* (**e**), *rimH* (**f**), *rimR*1 (**g**), and its own gene *rimR*2 (**h**). The 5′-biotin labeled DNA probe containing tested promoter regions were incubated with His_6_-tagged RimR2 protein. A 100-fold excess of unlabeled specific competitor was added to the competition assay, respectively. RimR2 protein binding putative promoter region of *rimA* gene **(a)**, *rimC* gene **(b)**, *rimD* gene **(c)**, *rimF* gene **(d)**, *rimG* gene **(d)**, *rimH* gene **(d)**, *rimR*1 gene **(g),** *rimR*2 gene **(h)**. The symbols “ + ” or “ − ”in the top row indicate the presence or absence of probes and competitors. Lane 1: biotin-labeled DNA probe; lane 2: biotin-labeled DNA probe plus RimR2 protein; lane 3: a 100-fold excess of unlabeled specific competitor plus RimR2 protein. All binding experiments were performed using 0.04 pmol/μl of biotin-labeled DNA probe and 10 μg of RimR2 protein

In vitro EMSA assay was performed to determine whether RimR2 could bind to the putative promoter region of the *rim* genes. In this experiment, His_6_-tagged RimR2 protein was generated in *E. coli* BL21 (DE3) (Additional file 5: Figure S4). The promoter region of each tested *rim* gene was designed as a biotin-labeled probe. Visible retarded bands were obtained for *rimR*2, *rimA* and *rimC*, whereas no retarded bands were obtained for the other tested promoter regions (Fig. 5). The binding specificity was assessed via the addition of excess unlabeled specific competitor. The 100-fold unlabeled probes strongly competed with the labeled probe to bind to *rimC*, and the retarded band was eliminated when specific unlabeled probes were added in excess. A similar phenomenon was observed in the case of RimR2 binding to the promoter region of *rimA* (Fig. 5). Moreover, this finding also indicates that RimR2 directly regulates its own transcription.

### Overexpression of *rimR*2 enhances rimocidin production

After establishing that RimR2 is responsible for rimocidin biosynthesis using deletion and complementation assays, we sought to increase rimocidin production by overexpressing *rimR*2 in *S. rimosus* M527. We assessed five different promoters: the constitutive promoter p*erm*E^*^, synthetic promoters pSPL21 and pSPL57, engineered promoter *kasO*p^*^, and its native promoter, and used them to drive *rimR*2 overexpression. The five corresponding recombinant plasmids were constructed (Additional file 6: Figure S5) and introduced into *S. rimosus* M527 by conjugation, yielding M527-ER, M527-21R, M527-57R, M527-KR, and M527-NR, respectively, which were resistant to 300 µg/ml apramycin (Additional file 7: Figure S6). PCR assays confirmed that the recombinant plasmids were integrated into the *S. rimosus* M527 chromosome (Additional file 8: Figure S7). These five recombinant strains and WT control were assessed via shake-flask fermentation.

**Fig. 6:**
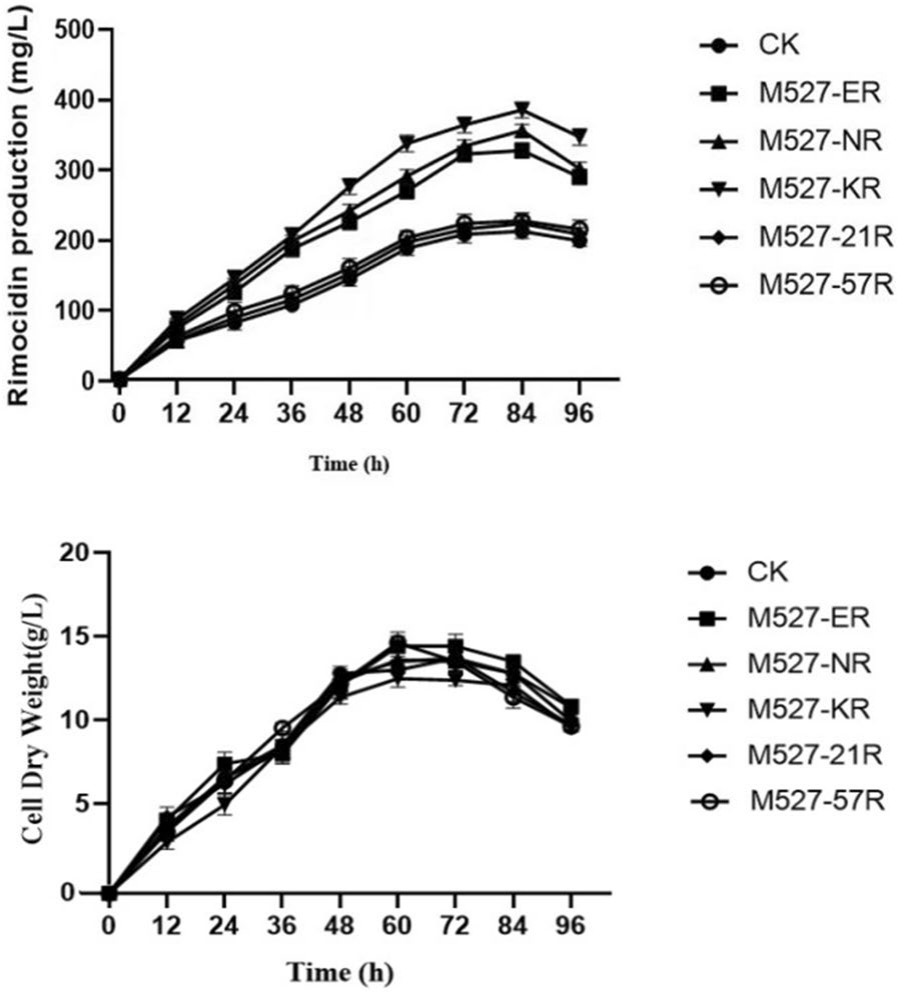
Detection and comparison of rimocidin production (**a**) and cell dry weight (**b**) of WT strain *S. rimosus* M527(●), recombinant strains M527-ER(■), M527-NR(▲), M527-KR(▼), M527-21R(◆) and M527-57R(○) in shake-flask culture experiment. All shake-flask fermentations were carried out in 250 ml flasks with a working volume of 40 ml at 200 rpm and 28 °C. The medium was inoculated at 5% (v/v). The error bars were calculated from three different batches of fermentation

M527-KR, M527-NR, and M527-ER produced more rimocidin than the control, with the highest amount being produced by M527-KR (376.7 mg/l), showing an 81.8% increase compared with the WT strain (207.2 mg/l) (Fig. 6). M527-NR (348.3 mg/l) and M527-ER (320.2 mg/l) produced 68.1% and 54.5% more rimocidin, respectively, and M527-21R and M527-57R showed no significant increase in rimocidin production compared with that of WT strain (Fig. 6a and Additional file 9: Figure S8). The overexpression of the *rimR*2 and integration of the empty vector pSET152 into the *S. rimosus* M527 genome did not significantly affect cell growth while the latter did not affect rimocidin production as well (Fig. 6b), consistent with the results of our previous study [37]. These results demonstrate that rimocidin production in *S. rimosus* M527 can be enhanced by overexpressing *rimR*2.

**Fig. 7:**
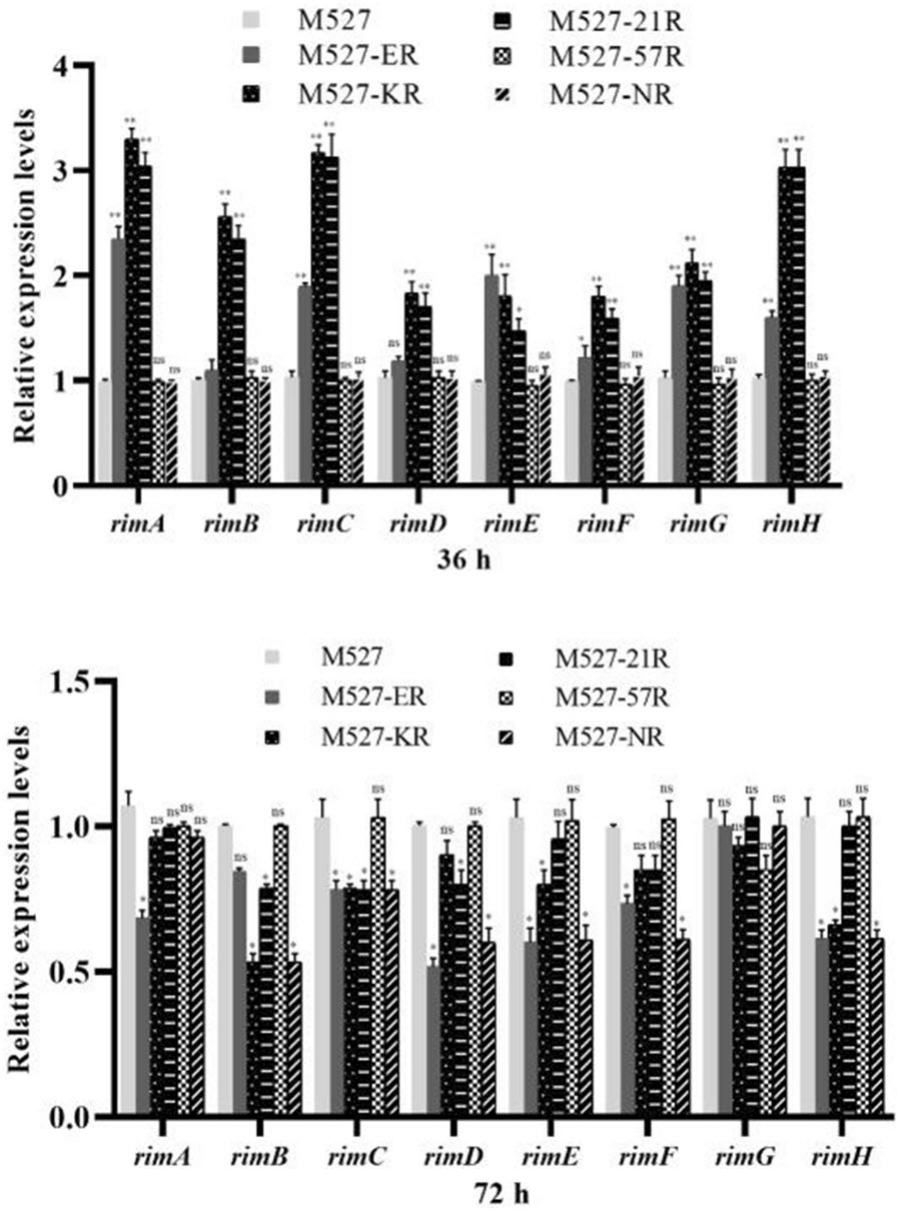
Comparison of the transcription levels of *rim* genes involved in rimocidin production in different strains obtained by quantitative reverse transcription-PCR (qRT-PCR). M527: *S. rimosus* M527; M527-KR: *S. rimosus* M527-KR; M527-NR: *S. rimosus* M527-NR; M527-ER: *S. rimosus* M527-ER; M527-21R: *S. rimosus* M527-21R; M527-57R: *S. rimosus* M527-57R. The cells were harvested from the fermentation broth after 36 and 72 h. Error bars were calculated by measuring the standard deviations of the data from three replicates of each sample. (^**^) indicates highly statistically significant results (*P*-value < 0.01)

The transcriptional levels of the *rim* genes in WT and recombinant strains following 36 and 72 h of fermentation were analyzed by using the qRT-PCR. As shown in (Fig. 7), the transcriptional level of all *rim* genes were up-regulated to varying degrees in M527-KR, M527-NR and M527-ER compared with that in the WT strain. These data combined with the EMSA assay results suggest that RimR2 directly activates the expression of *rimA* and *rimC* and indirectly activates the expression of the other *rim* genes.

## Discussion

Polyene compound biosynthesis is normally regulated by a pathway-specific regulator located in the biosynthetic gene cluster [39–43], however, the regulatory mechanism for rimocidin is yet to be identified. Recently, *S. rimosus* M527 was reported to be a major rimocidin producer [14]. In our previous study, we predicted and analyzed the rimocidin biosynthetic gene cluster in *S. rimosus* M527 using genome sequencing and antiSMASH. We discovered structural genes very similar to those reported by Seco et al. [38] in addition to four novel regulatory genes, *rimR*1-*rimR*4. In our earlier experiment, the genes *rimR*1*-rimR*4 were placed under the control of the *erm*E^*^ promoter in the plasmid pIB139 to create pIB139-*rimR*1*/*pIB139-*rimR*2*/*pIB139-*rimR*3 */*pIB139-*rimR*4 (Additional file 10: Figure S9). All four plasmids were introduced into *S. rimosus* M527 by intergeneric conjugation. The integration of the plasmids into the chromosome of *S. rimosus* M527 was verified via phenotypic and PCR analyses. The rimocidin productions of M527-R1, M527-R2 (M527-ER), M527-R3, and M527-R4 were determined via a shake-flask experiment (Additional file 11: Fig. S10a). After 84 h, the rimocidin yield of M527-R2 reached 320.2 mg/l, a 54.5% increase compared with that of the WT strain. The rimocidin yield of M527-R1 increased by approximately 20%-25%, whereas it did not differ significantly in M527-R4 compared with that of the WT strain. The overexpression of *rimR*3 exhibited a slightly negative effect on the rimocidin production (Additional file 11: Figure S10a). The difference in the rimocidin production of the recombinant strains was also reflected by the differently sized inhibition zones around the fungus *Fusarium oxysporum* f. sp. *cucumerinum*. M527-R2 demonstrated inhibition zones with larger diameters than those demonstrated by the WT strain (Additional file 11: Figure S10b). Therefore, the study on RimR2 has become a primary objective of this study. To elucidate its function, we deleted, complemented, and overexpressed *rimR*2 using different promoters in *S. rimosus* M527. We found that RimR2 belongs to the LAL subfamily of LuxR transcriptional regulators and plays a positive role in rimocidin biosynthesis.

RimR1 shares high similarity with several well-studied transcriptional regulators of the PAS-LuxR family, for example, it shares 49.61% amino acid sequence identity with PimM from *S. natalensis* AM493721.1 (Additional file 13: Figure S12). Phylogenetic tree analysis revealed that RimR2, RimR3, and RimR4 exhibit high sequence identity with certain well-studied LAL family regulators of the *Streptomyces* species (Additional file 14: Figure S13). Genes encoding PAS-LuxR regulators are present in almost all polyene macrolide biosynthetic gene clusters [43–45]. PimM, a member of the PAS-LuxR family, is a positive pathway-specific activator of pimaricin biosynthesis in *S. natalensis* [25]. Notably, PimM homologous regulatory proteins have been found to be encoded in the known polyene macrolide biosynthetic gene cluster, and all these regulators are functionally conserved [45]. Surprisingly, rimocidin production did not significantly increase with *rimR*1 overexpression. CTVGGGAWWTCCCBAG (where V is A, C, or G; W is A or T; and B is C, G, or T) is the consensus nucleotide sequence of the binding site of PAS-LuxR regulators has been revealed [45–47]. Using this sequence, we searched for a similar binding site in the rimocidin biosynthetic gene cluster in *S. rimosus* M527 and found three matches similar to the sixteen conserved nucleotide sequences: (1) CTAGGGAATTCCCGAG, which was the most similar to the consensus sequence. It is located 103-bp upstream of open reading frame 18, which encodes putative GDP-mannose 4,6-dehydratase, but does not lie within its promoter region; (2) GCCAGGAATTCCCGCA, situated near the 3′-end of the internal sequence of *rimF*, which encodes an aminotransferase, but does not lie within the putative promoter region of *rimG* encoding cytochrome P450 monooxygenase; (3) ACCGGAAAATCCTTAG, which is present in the intergenic region of *rimE* and *rimD,* 100-bp upstream of *rimE* but not within its putative promoter region*.* The locations of these three sequences suggest that they do not comprise the core elements for gene expression, which may explain the limited regulatory effect exerted by RimR1 on rimocidin production. The mechanism whereby RimR1 regulates structural genes in the rimocidin gene cluster will be elucidated in a future study.

The biosynthetic gene clusters encoding polyene macrolide antibiotics have been sequenced and multiple regulatory genes, usually organized in a hierarchical network, have been identified within them [11, 30, 31, 42]. For example, Santos-Aberturas et al. illustrated the hierarchical relationship between the SARP-LAL regulator PimR and the PAS-LuxR regulator PimM [31]. PimR stimulates pimaricin production by regulating PimM expression [31]. Herein, the EMSA assay demonstrated that RimR2 does not directly interact with the *rimR*1 promoter. Moreover, qRT-PCR revealed that *rimR*2 deletion decreased *rimR*1 expression and restored it to a level comparable with that in the WT strain M527 when it was complemented, suggesting that RimR2 indirectly regulates *rimR*1 expression. The relationship between RimR1 and RimR2 and their regulatory hierarchy are also worth investigating in a future study.

RimA serves as a loading module for rimocidin biosynthesis, and its upregulation is favorable for the overproduction of rimocidin [48]. Another tetraene that is a structural analog of rimocidin, CE-108, was also found in the fermentation broth of *S. rimosus* M527. These two tetraenes differ in the aglycone moiety, with a propyl group in rimocidin and a methyl group in CE-108. As the elongation module is common for both rimocidin and CE-108 biosyntheses, RimR2 regulates both biosyntheses almost identically (Additional file 9: Figure S8). However, CE-108 exhibits a weaker antifungal activity than rimocidin [8]. Therefore, a strategy to specifically increase rimocidin production is worth devising in the future.

To improve rimocidin production in *S. rimosus* M527, five different promoters were assessed to overexpress *rimR*2. As the WT strain contains only a single *attB* attachment site, the five recombinant plasmids were derived from the pSET152 backbone. The M527-ER strain, wherein *rimR*2 expression was driven by the p*erm*E^*^ promoter, produced 75% more rimocidin than that produced by the WT. The engineered promoter *kasO*p^*^, which exhibits higher activity than the promoter p*erm*E^*^ in some streptomycetes, was used in *S. rimosus* M527 for the first time and found that it optimally enhanced rimocidin yield. M527-KR produced 15% more rimocidin than M527-ER, indicating that *kasO*p^*^ is more effective than the promoter p*erm*E^*^ for gene expression in *S. rimosus* M527. Because RimR2 self-regulates its own promoter, M527-NR harboring the 3057-bp *rimR*2 gene with its own 300-bp promoter also exhibits higher rimocidin production than the WT strain. Surprisingly, despite their higher activity than p*erm*E^*^ in *S. rimosus* M527, SPL21- and SPL57-driven *rimR*2 overexpression did not significantly increase rimocidin yield. For the optimal efficacy of a promoter, its expression strength must match the cell growth and metabolic flux.

In conclusion, RimR2 is the first pathway-specific transcriptional regulator of rimocidin biosynthesis to be described to date. Our results reveal that *rimR*2, found in the rimocidin biosynthetic gene cluster, encodes a positive regulatory protein that strongly influences rimocidin production by controlling the transcription of structural genes. Furthermore, overexpressing *rimR*2 could promote rimocidin production. Further studies are warranted to explore the core sequence of the promoter to which RimR2 binds. The long-term goal of our research is to elucidate the exact mechanism underlying the regulation of rimocidin biosynthesis to improve its yield using rational metabolic engineering. In addition, the acyl-coenzyme A transferase domain of RimA can be engineered to enhance the amount of rimocidin produced at the expense of its structural analog, CE-108.

## Materials and methods

### Materials

Chemicals, biochemicals, molecular biology reagents, endonucleases, and different kits were purchased from standard commercial sources.

### Strains, plasmids, and primers

The strains and plasmids used in this study are listed in Table 1, and the primers are listed in Additional file 15: Table S2.

**Table 1:** The strains and plasmids used in this study

Strains/plasmids	Description	Source
Strains		
*E. coli* JM109	General cloning host	Our lab
*E. coli* ET12567 (pUZ8002)	*Cm*^r^, *Km*^r^, donor strain for conjugation	Our lab
*E. coli* BL21(DE3)	Strain for protein expression and purification	Our lab
*S. rimosus* M527	Parental strain, rimocidin producer	CCTCC M2013270
M527-ER	M527 containing integrative vector pIB139-*rimR*2	This work
M527-KR	M527 containing integrative vector pSET152-KR	This work
M527-21R	M527 containing integrative vector pSET152-21R	This work
M527-57R	M527 containing integrative vector pSET152-57R	This work
M527-NR	M527 containing integrative vector pSET152::*rimR*2	This work
M527-pSET152	M527 containing integrative vector pSET152	This work
M527-ΔrimR2	*rimR*2 gene disruption mutant, derived from M527 strain	This work
M527-ΔrimR2/ pSET152	Mutant M527-ΔrimR2 with integrative plasmid pSET152	This work
M527-ΔrimR2/ pSET152::*rimR*2	*rimR*2 complemented strain, mutant M527-ΔrimR2 with integrative plasmid pSET152::*rimR*2	This work
Plasmids		
pIB139	*Ap*^r^, *perm*E^*^, integrative vector, based on pSET152	Our lab
pSET152	*Ap*^r^, no promoter, integrative vector	Our lab
pWHU2653	Scas9, sgRNA cloning cassette*, codA(sm), apr*^*r*^, *ori(coE*l)	Zeng et al. [49]
pGUS-SPL21	*gusA* under the control of promoter SPL21	Siegl et al. [50]
pGUS-SPL57	*gusA* under the control of promoter SPL57	Siegl et al. [50]
pGUS-ermE^*^	*gusA* under the control of synthetic promoter p*erm*E^*^	Prof. Luzhetskyy
pDR4	*Hyg*^r^, *kasO*p^*^, based on pSET152	Wang et al. [51]
pSPL21-*rimR*2	Derived from pGUS-SPL21, *gusA* was replaced by *rimR*2 gene	This work
pSPL57-*rimR*2	Derived from pGUS-SPL57, *gusA* was replaced by *rimR*2 gene	This work
p*erm*E^*^-*rimR*2	Derived frompGUS-ermE^*^, *gusA* was replaced by *rimR*2 gene	This work
pDR4-*rimR*2	Derived from pDR4, *rimR*2 gene under the control of *kasO*p^*^	This work
pSET152::*rimR*2	Derived frompSET152, harboring *rimR*2 gene driven by its own promoter	This work
pET32a	*Amp*^r^, Expression vector	Our lab
pET32a-*rimR*2	Derived from pET32a, harboring *rimR*2 gene driven by T_7_ promoter	This work

The promoters permE* and kasOp* were mutated or engineered from native promoter, which were reported and usually used for gene over-expression in streptomycetes

### Media and culture conditions

The media and culture conditions of *E. coli*, *S. rimosus* M527 and its derivatives were described by Zhao et al. [36].

### DNA manipulations

DNA extraction and manipulation in *E. coli* were conducted following the standard protocol as described by Sambrook and Russel [52]. Genetic manipulations and intergeneric conjugation of *Streptomyces* were performed according to the standard protocol as described by Kieser et al. [53].

### Construction of *rimR*2 disruption (ΔrimR2) mutant and its complementation

According to the previously described methods [49, 54], the disruption of the *rimR*2 gene was performed by using the CRISPR/Cas9. In terms of plasmid construction, the 0.3-kb sgRNA cloning cassette was inserted into pWHU2653 between *Nhe* I/*Xba* I using an infusion cloning kit, generating plasmid pWHU2653-sgRNA. Subsequently, using *S. rimosus* M527 genomic DNA as a template, the 2.0-kb upstream homologous arm of the *rimR*2 start codon and 2.0-kb downstream homologous arm of the *rimR*2 stop codon were amplified by using primer pairs P1/P2 and P3/P4, respectively, and ligated into the *Hin*d III site of pWHU2653-sgRNA using Gibson assembly methods as described by Gibson et al. [55], yielding plasmid pWHU2653-Δ*rimR*2 for gene knockout.

The constructed pWHU2653-Δ*rimR*2 was transferred into the WT strain *S. rimosus* M527 from *E. coli* ET12567/pUZ8002 by intergeneric conjugation, following the method as described by Song et al. [56]. To determine CRISPR/Cas9 mediated recombination, each selected apramycin sensitive colony was subjected to PCR using primers (PrimR-F1/R1). The gene-deleted mutant was designated *S. rimosus* M527-ΔrimR2.

Construction of plasmid pSET152::*rimR*2 harboring the coding region of *rimR*2 gene and its 300-bp promoter sequence was presented in Additional file 6: Figure S5. According to previous methods as described by Liao et al. [54], a similar procedure was adopted and performed to generate complemented strain *S. rimosus* M527-ΔrimR2/pSET152::*rimR*2. As a control, the empty vector pSET152 was also introduced into mutant M527-ΔrimR2 by conjugation to generate strain M527-ΔrimR2/pSET152.

### Overexpression of the *rimR*2 gene in *S. rimosus* M527

According to previous methods as described by Xu et al. [57], a similar procedure was adopted and conducted to generate plasmids pSPL21-*rimR*2, pSPL57*-rimR*2, p*erm*E^*^-*rimR*2 and pDR4-*rimR*2 (Additional file 6: Figure S5)*.* Primers PrimR2-F1/R1/R2 (Additional file 15: Table S2) were used to amplified *rimR*2 gene for plasmid construction.

The five constructed plasmids pSPL21-*rimR*2, pSPL57*-rimR*2, p*erm*E^*^-*rimR*2, pDR4-*rimR*2, and pSET152::*rimR*2 together with the control empty vector pSET152 were introduced into *S. rimosus* M527 from *E. coli* ET12567/pUZ8002 by conjugation to generate recombinant strains *S. rimosus* M527-21R, M527-57R, M527-ER, M527-KR, and M527-NR, respectively. Phenotypic and genotypic verification of exconjugants was based on the selection of apramycin resistance and amplification of the apramycin resistance gene, respectively.

### Analysis of *rim* genes transcriptional levels by using qRT-PCR

RNA extraction and the analysis of the transcriptional level of *rim* genes in the WT strain, mutant, complemented strain, and recombinant strains were performed as described by Zhao et al. [36]. qRT-PCR primers Y_rimR1_F (5′-GGAGTATCACGTCACCGGAC-3′) and Y_rimR1_R (5′-GATGAAGCCCTCGACGACAC-3′) were designed following the *rimR*1 gene sequence (GenBank accession no. MK300953).

### Expression and purification of RimR2 protein

A 2757-bp DNA fragment harboring the *rimR*2 coding sequence was amplified by PCR using primers PrimR2-F2/R3 (Additional file 15: Table S2) and *S. rimosus* M527 genomic DNA as a template. The *rimR*2 gene was inserted into the *Eco*R V and *Bam*H I sites of pET32a to generate the plasmid pET32a-*rimR*2. Then, plasmid pET32a-*rimR*2 was expressed in *E. coli* BL21 (DE3). RimR2 protein was purified using nickel-NTA column (Qiagen) and eluted using imidazole. The inducible expression and purification of were performed according to standard manipulation method described by Sambrook and Russel [52].

### Electrophoretic mobility-shift assays (EMSA)

The putative promoter regions of the *rim* genes were amplified by PCR using the biotin labeled primers (Additional file 16: Table S3) and *S. rimosus* M527 genomic DNA as a template. The biotin-labeled probe, and an unlabeled probe as competitors (Additional file 16: Table S3) were also used. Except for the addition of biotin at the 3’ end of the sequence, the sequence of the biotin-labeled probe was identical to that of the competitive probe. EMSAs were performed using a Light shift Chemiluminescent EMSA Kit (Thermo, Fisher, MA, USA) according to the manufacture’s protocol. The biotinylated probes were transferred to a nylon membrane (Millipore). Blocking with 15 ml blocking buffer for 15 min, and adding 50 μl stabilized Streptavidin-HRP for 15 min every 15 ml blocking buffer were conducted. Then it was washed four times using wash buffer (1 ×), each time for five minutes, and finally the balancing solution substrate equilibration buffer was used. The membranes were dried and exposed to UV radiation to cross-link the DNA fragments. Finally, protein-bound and free DNAs were detected by chemiluminescence, and the signals were recorded on X-ray film.

### Production and HPLC analysis of rimocidin

Fermentation of rimocidin and HPLC analysis of fermentation broth were performed as described by Zhao et al. [36]. The presence of rimocidin was analyzed and confirmed using HPLC with a column of Supersil ODS2 (4.6 × 150 mm, 5 μm) maintained at 30 °C. The percentage volume of methanol was varied as follows: linearly increased from 5 to 83% (0–20 min), held at 83% (20–30 min), linearly increased to 100% (30–35 min), and then linearly decreased to 5% (35–40 min). The UV detection of rimocidin was conducted at 304 nm and the solvent flow rate was 1.0 mL/min.

### Bioinformatics analysis

The genome was mined using bioinformatic tool (antiSMASH) for the identification of clusters involved in rimocidin, and function annotation of biosynthetic gene cluster was listed in https://fungismash.secondarymetabolites.org/upload/fungi-849fa284-178d-4d88-80cb-6bfd37f228f7/index.html#r75c1. The promoters of rimocidin biosynthetic gene cluster were predicted on website http://nucleix.mbu.iisc.ernet.in/prompredict/prompredict.html.

### Statistical analysis

All experiments were carried out at least three times, and the results were expressed by the mean ± standard deviation (SD). Students’ *t* test was used for statistical analysis.

## Supplementary Information

**Additional file 1: Table S1.** Detailed information of RimR2 and some polyene macrolide biosynthesis regulators from other* Streptomyces* species in phylogenetic tree.

**Additional file 2: Figure S1.** Construction of mutant* S. rimosus* M527-ΔrimR2. Map of plasmid pWHU2653- Δ*rimR*2. The sgRNA consists of the 20 nt target gene specific guide sequence of *S. rimosus* M527 (green) and the invariant scaffold RNA (yellow). Light blue parallelograms connect the identical UHA and DHA sequences on pWHU2653 and the *S. rimosus* M527 chromosome where homologous recombination can take place.

**Additional file 3: Figure S2.** PCR verification of the mutant *S. rimosus *M527-ΔrimR2. M: DL5000 DNA Marker. Lane 1, The PCR products of 2.8-kb *rimR*2 gene were amplified by using the primers PrimR2-F1/R1 from WT strain *S. rimosus *M527; Lane 2, The PCR products of 6.8-kb cassette containing 2.8-kb *rimR*2 gene and its 2.0-kb upstream and 2.0-kb downstream fragment were amplified by using the primers P1/P4 from *S. rimosus *M527; Lane 3-5, The PCR products of *rimR*2 gene were amplified by using the PrimR2-F1/R1 from three randomly mutant strains M527-ΔrimR2; Lane 6-8, The PCR products of 4.0-kb cassette containing 2.0-kb upstream and 2.0-kb downstream fragment were amplified by using the P1/P4 from three randomly mutant strains M527-ΔrimR2.

**Additional file 4: Figure S3.** HPLC analysis of rimocidin production in the WT strain *S. rimosus *M527, in mutant *S. rimosus *M527-ΔrimR2, and in the complemented strain *S. rimosus *M527-ΔrimR2/pSET152::*rimR*2*, *and control strain *S. rimosus *M527/pSET152.

**Additional file 5: Figure S4.** Purification and elution of RimR2 protein. M: Protein Marker; Lane 1, Purified His6-tagged RimR2 protein after affinity nickel-NTA column. Lanes 2-4, Eluted RimR2 protein with 250 mM, 300mM, 500mM imidazole.

**Additional file 6: Figure S5.** Construction of recombinant plasmids for over-expression of *rimR*2 gene with different promoters.

**Additional file 7: Figure S6.** Phenotypic verification of recombinant strains recombinant strains harboring over-expression of *rimR*2 gene. Recombinant strains could grow on 2CMC agar medium containing 300 μg/ml apramycin, while control strain *S. rimosus* M527 did not. 2CMC agar medium was incubated at 28 °C for 4 days.

**Additional file 8: Figure S7.** PCR analysis of apramycin (*ap*^*r*^) gene from recombinant strains harboring over-expression of *rimR* gene. DL DNA 2000 marker was used (M). Lane 1: PCR product of *ap*^*r*^ gene from *S. rimosus* M527(negative control); lane 2: PCR product of *ap*^*r*^ gene from plasmid pSET152(positive control); lane 3-5: PCR product of *ap*^*r*^ gene from recombinant strains *S. rimosus* M527-ER; lane 6-8: PCR product of *ap*^*r*^ gene from recombinant strains *S. rimosus* M527-KR; lane 9-11: PCR product of *ap*^*r*^ gene from recombinant strains *S. rimosus* M527-NR; lane 12-14: PCR product of *ap*^*r*^ gene from recombinant strains *S. rimosus* M527-21R; lane 15-17: PCR product of *ap*^*r*^ gene from recombinant strains *S. rimosus* M527-57R.

**Additional file 9: Figure S8.** HPLC analysis of rimocidin isolated from fermentation extracts of the recombinant strains *S. rimosus* M527-KR, *S. rimosus* M527-NR, *S. rimosus* M527-ER, *S. rimosus* M527-21R, *S. rimosus* M527-57R and WT strain *S. rimosus* M527.

**Additional file 10: Figure S9.** Construction of recombinant plasmids of overexpression of *rimR*1*/rimR*2*/rimR*3 */rimR*4 gene with p*erm*E^*^ promoter.

**Additional file 11: Figure S10. **Detection and comparison of rimocidin production (**a**) and cell dry weight (**b**) of WT strain *S. rimosus* M527(●), recombinant strains M527-R1(■), M527-R2(▲), M527-R3(▼), and M527-R4(◆) in shake-flask culture experiment.

**Additional file 12: Figure S11.** Detection and comparison of antifungal activities of WT strain M527, recombinant strains M527-R1, M527-R2, M527-R3, and M527-R4 against *F. oxysporum *f. sp. *cucumerinum*. Spore suspension (500 μl) of *F. oxysporum* f. sp*. cucumerinum* (1×10^6^ cfu ml^-1^) was spread and inoculated on PDA medium at 28 °C for 1 d. A agar block (4 mm in diameter) containing actively growing WT strain M527, three random recombinant strains M527-R1(**a**), M527-R2(**b**), M527-R3(**c**), and M527-R4(**d**) was aseptically placed on aforementioned PDA medium containing pathogenic fungus at 28 °C for 3-4 d. The diameter of inhibition zone was measured as antagonistic activity. Plant-pathogenic fungus *F. oxysporum *f. sp. *cucumerinum* was used as indicator strain in antifungal activities assay.

**Additional file 13: Figure S12.** Phylogenetic tree of RimR1 and other polyene macrolide biosynthesis regulators (PAS-LuxR).

**Additional file 14: Figure S13.** Phylogenetic tree of RimR2, RimR3, RimR4 and other polyene macrolide biosynthesis regulators (LAL).

**Additional file 15: Table S2.** The primers used for deletion or expression of* rimR*2 gene in this study.

**Additional file 16: Table S3.** The primers used for EMSA assay in this study.

## Data Availability

All data generated or analyzed during this study are included in this published article [and its Additional files].

## Abbreviations

PKSs: Polyketide synthases
PEM: Polyene macrolide antibiotics
WT: Wide-type
EMSA: Electrophoretic mobility shift assays
qRT-PCR: Quantitative RT-PCR
sgRNA: Single guide RNA
CCTCC: China Center for Type Culture Collection
CGMCC: China General Microbiological Culture Collection Center
PAM: Protospacer adjacent motif
UHA: Upstream homologous arm
DHA: Downstream homologous arm
ORF: Open reading frame
HPLC: High-performance liquid chromatography
SD: Standard deviations
CRISPR/Cas: Clustered regularly interspaced short palindromic repeats(CRISPR) /CRISPR-associated
SARP: *Streptomyces* antibiotic regulatory protein
HTH: Helix-turn-helix
LAL: Larger ATP-binding regulators of the LuxR
