# Supplementary material for: Identification of RimR2 as a positive pathway-specific regulator of rimocidin biosynthesis in Streptomyces rimosus M527
Source: Microb Cell Fact. 2023 Feb 21;22:32. doi: 10.1186/s12934-023-02039-9 (PMC9942304; doi:10.1186/s12934-023-02039-9)
Supplement: Supplementary file 1 — Additional file 1: Table S1. Detailed information of RimR2 and some polyene macrolide biosynthesis regulators from other Streptomyces species in phylogenetic tree. [file 12934_2023_2039_MOESM1_ESM.docx]

**Additional file 1:**

**Table S1** Detailed information of RimR2 and some polyene macrolide biosynthesis regulators from other *Streptomyces* species in phylogenetic tree.

| **Strains** | **Polyene macrolide** | **Regulators** | **Type of regulators** | **GenBank Accession number** |
| --- | --- | --- | --- | --- |
| *Streptomyces rimosus* | Rimocidin | RimR2 | LAL | QAS68949.1 |
| *Streptomyces nodosus* | Amphotericin | AmphRI | LAL | AAV37059.1 |
| *Streptomyces nodosus* | Amphotericin | AmphRII | LAL | AAV37060.1 |
| *Streptomyces nodosus* | Amphotericin | AmphRIII | LAL | AAV37061.1 |
| *Streptomyces nodosus* | Amphotericin | AmphRIV | PAS-LuxR | AAV37062.1 |
| *Streptomyces nodosus* | Amphotericin | AmphRVI | LAL | AJE44551.1 |
| *Streptomyces aureofuscus* | Anreofuscin | AURJ3M | PAS-LuxR | ACD75765.1 |
| *Streptomyces avermitilis* | Filipin | PteR | SAPR-LAL | BAB69312.1 |
| *Streptomyces avermitilis* | Filipin | PteF | PAS-LuxR | BAC68119.1 |
| *Streptomyces filipinensis* | Filipin | FilF | PAS-LuxR | AKX77828.1 |
| *Streptomyces filipinensis* | Filipin | FilR | SAPR-LAL | AKX77827.1 |
| *Streptomyces* sp. FR-008 | Candicidin | FscRI | PAS-LuxR | AAQ82551.1 |
| *Streptomyces* sp. FR-008 | Candicidin | FscRII | LAL | AAQ82552.1 |
| *Streptomyces* sp. FR-008 | Candicidin | FscRIII | LAL | AAQ82553.1 |
| *Streptomyces* sp. FR-008 | Candicidin | FscRIV | LAL | AAQ82554.1 |
| *Streptomyces noursei* | Nystatin | NysRI | LAL | AAF71778.1 |
| *Streptomyces noursei* | Nystatin | NysRII | LAL | AAF71779.1 |
| *Streptomyces noursei* | Nystatin | NysRIII | LAL | AAF71780.1 |
| *Streptomyces noursei* | Nystatin | NysRIV | PAS-LuxR | AAF71781.1 |
| *Streptomyces chattanoogensis* | Natamycin | ScnRI | SAPR-LAL | ADX66458.1 |
| *Streptomyces chattanoogensis* | Natamycin | ScnRII | PAS-LuxR | ADX66474.1 |
| *Streptomyces ahygroscopicus* | Tetramycin | TtmRI | LAL | AFW98290.1 |
| *Streptomyces ahygroscopicus* | Tetramycin | TtmRII | LAL | AFW98288.1 |
| *Streptomyces ahygroscopicus* | Tetramycin | TtmRIII | LAL | AFW98289.1 |
| *Streptomyces ahygroscopicus* | Tetramycin | TtmRIV | PAS-LuxR | AFW98287.1 |
| *Streptomyces natalensis* | Natamycin | PimM | PAS-LuxR | AM493721.1 |
| *Streptomyces natalensis* | Natamycin | PimR | SAPR-LAL | CAE51066.1 |
