# Supplementary material for: Identification of RimR2 as a positive pathway-specific regulator of rimocidin biosynthesis in Streptomyces rimosus M527
Source: Microb Cell Fact. 2023 Feb 21;22:32. doi: 10.1186/s12934-023-02039-9 (PMC9942304; doi:10.1186/s12934-023-02039-9)

**Additional file 2:**

**Figure S1.** Construction of mutant *S. rimosus* M527-ΔrimR2. Map of plasmid pWHU2653-Δ*rimR*2. The sgRNA consists of the 20 nt target gene specific guide sequence of *S. rimosus* M527 (green) and the invariant scaffold RNA (yellow). Light blue parallelograms connect the identical UHA and DHA sequences on pWHU2653 and the *S. rimosus* M527 chromosome where homologous recombination can take place.


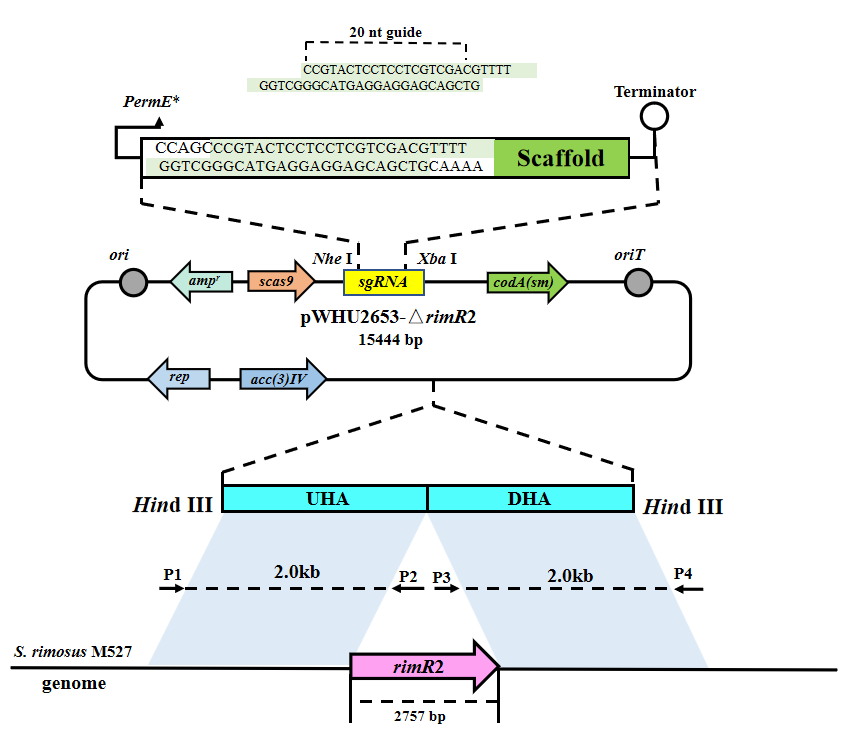

Supplement: Supplementary file 2 — Additional file 2: Figure S1. Construction of mutant S. rimosus M527-ΔrimR2. Map of plasmid pWHU2653- ΔrimR2. The sgRNA consists of the 20 nt target gene specific guide sequence of S. rimosus M527 (green) and the invariant scaffold RNA (yellow). Light blue parallelograms connect the identical UHA and DHA sequences on pWHU2653 and the S. rimosus M527 chromosome where homologous recombination can take place. [file 12934_2023_2039_MOESM2_ESM.docx]
