# Supplementary material for: Identification of RimR2 as a positive pathway-specific regulator of rimocidin biosynthesis in Streptomyces rimosus M527
Source: Microb Cell Fact. 2023 Feb 21;22:32. doi: 10.1186/s12934-023-02039-9 (PMC9942304; doi:10.1186/s12934-023-02039-9)

**Additional file 3:**

**Figure S2.** PCR verification of the mutant *S. rimosus* M527-ΔrimR2. M: DL5000 DNA Marker. Lane 1, The PCR products of 2.8-kb *rimR*2 gene were amplified by using the primers PrimR2-F1/R1 from WT strain *S. rimosus* M527; Lane 2, The PCR products of 6.8-kb cassette containing 2.8-kb *rimR*2 gene and its 2.0-kb upstream and 2.0-kb downstream fragment were amplified by using the primers P1/P4 from *S. rimosus* M527; Lane 3-5, The PCR products of *rimR*2 gene were amplified by using the PrimR2-F1/R1 from three randomly mutant strains M527-ΔrimR2; Lane 6-8, The PCR products of 4.0-kb cassette containing 2.0-kb upstream and 2.0-kb downstream fragment were amplified by using the P1/P4 from three randomly mutant strains M527-ΔrimR2.


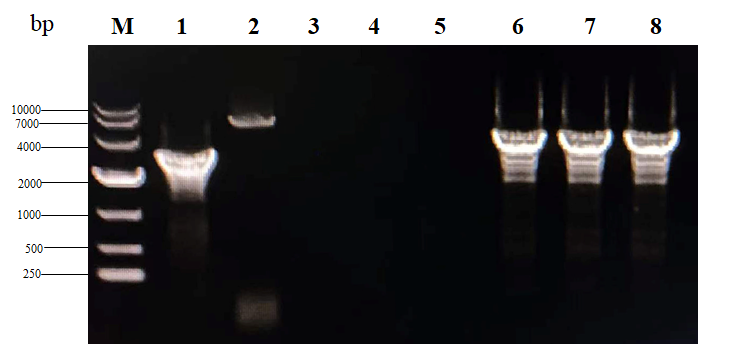

Supplement: Supplementary file 3 — Additional file 3: Figure S2. PCR verification of the mutant S. rimosus M527-ΔrimR2. M: DL5000 DNA Marker. Lane 1, The PCR products of 2.8-kb rimR2 gene were amplified by using the primers PrimR2-F1/R1 from WT strain S. rimosus M527; Lane 2, The PCR products of 6.8-kb cassette containing 2.8-kb rimR2 gene and its 2.0-kb upstream and 2.0-kb downstream fragment were amplified by using the primers P1/P4 from S. rimosus M527; Lane 3-5, The PCR products of rimR2 gene were amplified by using the PrimR2-F1/R1 from three randomly mutant strains M527-ΔrimR2; Lane 6-8, The PCR products of 4.0-kb cassette containing 2.0-kb upstream and 2.0-kb downstream fragment were amplified by using the P1/P4 from three randomly mutant strains M527-ΔrimR2. [file 12934_2023_2039_MOESM3_ESM.docx]
