# Supplementary material for: Identification of RimR2 as a positive pathway-specific regulator of rimocidin biosynthesis in Streptomyces rimosus M527
Source: Microb Cell Fact. 2023 Feb 21;22:32. doi: 10.1186/s12934-023-02039-9 (PMC9942304; doi:10.1186/s12934-023-02039-9)

**Additional file 4:**

**Figure S3.** HPLC analysis of rimocidin production in the WT strain *S. rimosus* M527, in mutant *S. rimosus* M527-ΔrimR2, and in the complemented strain *S. rimosus* M527-ΔrimR2/pSET152::*rimR*2*,* and control strain *S. rimosus* M527/pSET152.


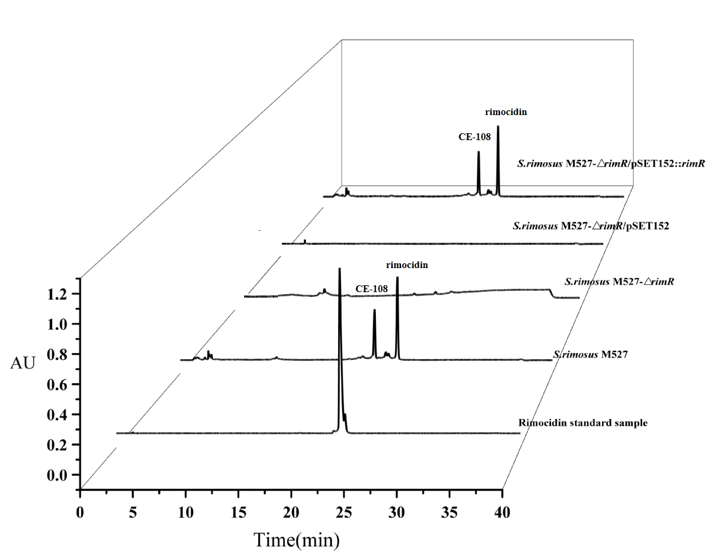

Supplement: Supplementary file 4 — Additional file 4: Figure S3. HPLC analysis of rimocidin production in the WT strain S. rimosus M527, in mutant S. rimosus M527-ΔrimR2, and in the complemented strain S. rimosus M527-ΔrimR2/pSET152::rimR2, and control strain S. rimosus M527/pSET152. [file 12934_2023_2039_MOESM4_ESM.docx]
