# Supplementary material for: Identification of RimR2 as a positive pathway-specific regulator of rimocidin biosynthesis in Streptomyces rimosus M527
Source: Microb Cell Fact. 2023 Feb 21;22:32. doi: 10.1186/s12934-023-02039-9 (PMC9942304; doi:10.1186/s12934-023-02039-9)

**Additional file 5:**

**Figure S4.** Purification and elution of RimR2 protein. M: Protein Marker; Lane 1, Purified His6-tagged RimR2 protein after affinity nickel-NTA column. Lanes 2-4, Eluted RimR2 protein with 250 mM, 300mM, 500mM imidazole.


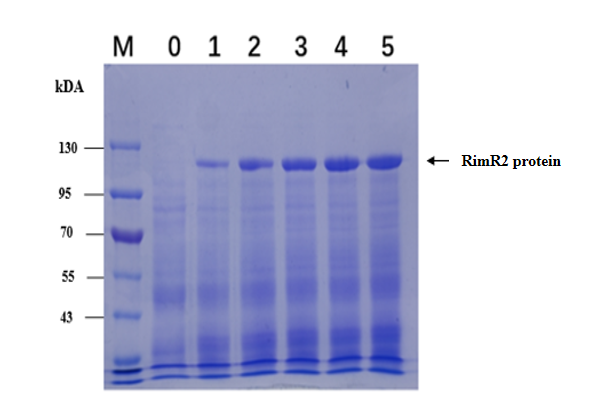

Supplement: Supplementary file 5 — Additional file 5: Figure S4. Purification and elution of RimR2 protein. M: Protein Marker; Lane 1, Purified His6-tagged RimR2 protein after affinity nickel-NTA column. Lanes 2-4, Eluted RimR2 protein with 250 mM, 300mM, 500mM imidazole. [file 12934_2023_2039_MOESM5_ESM.docx]
