# Supplementary material for: Identification of RimR2 as a positive pathway-specific regulator of rimocidin biosynthesis in Streptomyces rimosus M527
Source: Microb Cell Fact. 2023 Feb 21;22:32. doi: 10.1186/s12934-023-02039-9 (PMC9942304; doi:10.1186/s12934-023-02039-9)

**Additional file 6:**

**Figure S5.** Construction of recombinant plasmids for over-expression of *rimR*2 gene with different promoters.


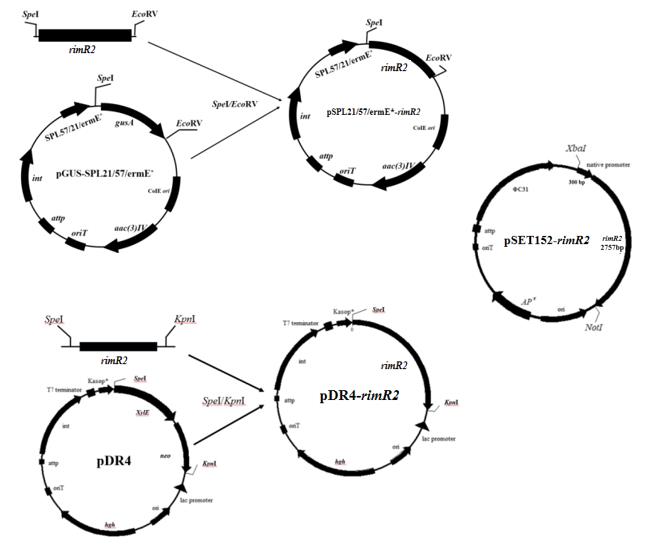

Supplement: Supplementary file 6 — Additional file 6: Figure S5. Construction of recombinant plasmids for over-expression of rimR2 gene with different promoters. [file 12934_2023_2039_MOESM6_ESM.docx]
