# Supplementary material for: Identification of RimR2 as a positive pathway-specific regulator of rimocidin biosynthesis in Streptomyces rimosus M527
Source: Microb Cell Fact. 2023 Feb 21;22:32. doi: 10.1186/s12934-023-02039-9 (PMC9942304; doi:10.1186/s12934-023-02039-9)
Supplement: Supplementary file 7 — Additional file 7: Figure S6. Phenotypic verification of recombinant strains recombinant strains harboring over-expression of rimR2 gene. Recombinant strains could grow on 2CMC agar medium containing 300 μg/ml apramycin, while control strain S. rimosus M527 did not. 2CMC agar medium was incubated at 28 °C for 4 days. [file 12934_2023_2039_MOESM7_ESM.docx]

**Additional file 7:**

**Figure S6.** Phenotypic verification of recombinant strains harboring over-expression of *rimR*2 gene. Recombinant strains could grow on 2CMC agar medium containing 300 μg/ml apramycin, while control strain *S. rimosus* M527 did not. 2CMC agar medium was incubated at 28 °C for 4 days.

**
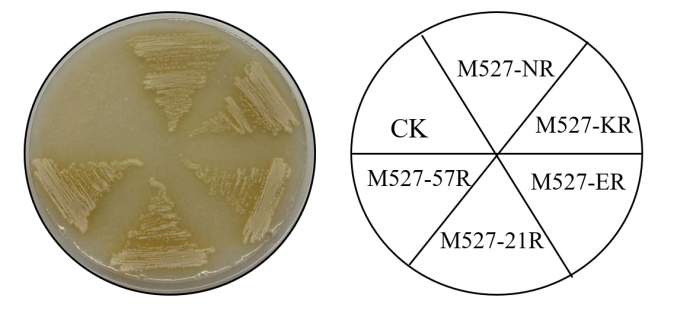
**
