# Supplementary material for: Identification of RimR2 as a positive pathway-specific regulator of rimocidin biosynthesis in Streptomyces rimosus M527
Source: Microb Cell Fact. 2023 Feb 21;22:32. doi: 10.1186/s12934-023-02039-9 (PMC9942304; doi:10.1186/s12934-023-02039-9)

**Additional file 8:**

**Figure S7.** PCR analysis of apramycin (*ap^r^*) gene from recombinant strains harboring over-expression of *rimR*2 gene. DL DNA 2000 marker was used (M). Lane 1: PCR product of *ap^r^* gene from *S. rimosus* M527(negative control); lane 2: PCR product of *ap^r^* gene from plasmid pSET152(positive control); lane 3-5: PCR product of *ap^r^* gene from recombinant strains *S. rimosus* M527-ER; lane 6-8: PCR product of *ap^r^* gene from recombinant strains *S. rimosus* M527-KR; lane 9-11: PCR product of *ap^r^* gene from recombinant strains *S. rimosus* M527-NR; lane 12-14: PCR product of *ap^r^* gene from recombinant strains *S. rimosus* M527-21R; lane 15-17: PCR product of *ap^r^* gene from recombinant strains *S. rimosus* M527-57R.


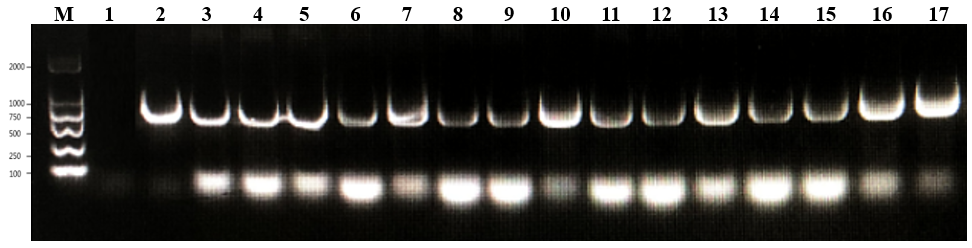

Supplement: Supplementary file 8 — Additional file 8: Figure S7. PCR analysis of apramycin (apr) gene from recombinant strains harboring over-expression of rimR gene. DL DNA 2000 marker was used (M). Lane 1: PCR product of apr gene from S. rimosus M527(negative control); lane 2: PCR product of apr gene from plasmid pSET152(positive control); lane 3-5: PCR product of apr gene from recombinant strains S. rimosus M527-ER; lane 6-8: PCR product of apr gene from recombinant strains S. rimosus M527-KR; lane 9-11: PCR product of apr gene from recombinant strains S. rimosus M527-NR; lane 12-14: PCR product of apr gene from recombinant strains S. rimosus M527-21R; lane 15-17: PCR product of apr gene from recombinant strains S. rimosus M527-57R. [file 12934_2023_2039_MOESM8_ESM.docx]
