# Supplementary material for: Identification of RimR2 as a positive pathway-specific regulator of rimocidin biosynthesis in Streptomyces rimosus M527
Source: Microb Cell Fact. 2023 Feb 21;22:32. doi: 10.1186/s12934-023-02039-9 (PMC9942304; doi:10.1186/s12934-023-02039-9)

**Additional file 9:**

**Figure S8.** HPLC analysis of rimocidin isolated from fermentation extracts of the recombinant strains *S. rimosus* M527-KR, *S. rimosus* M527-NR, *S. rimosus* M527-ER, *S. rimosus* M527-21R, *S. rimosus* M527-57R and WT strain *S. rimosus* M527.


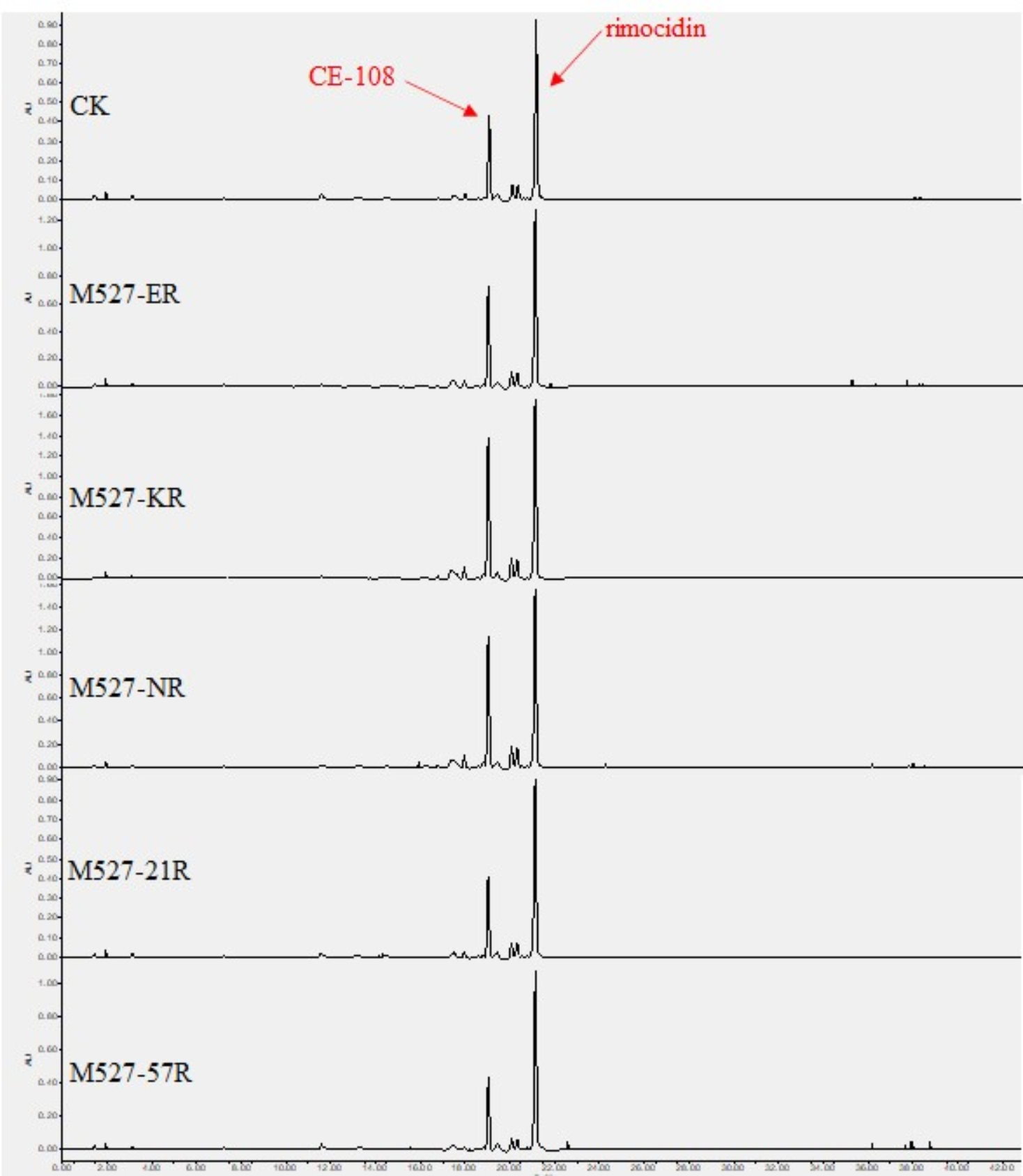

Supplement: Supplementary file 9 — Additional file 9: Figure S8. HPLC analysis of rimocidin isolated from fermentation extracts of the recombinant strains S. rimosus M527-KR, S. rimosus M527-NR, S. rimosus M527-ER, S. rimosus M527-21R, S. rimosus M527-57R and WT strain S. rimosus M527. [file 12934_2023_2039_MOESM9_ESM.docx]
