# Supplementary material for: Identification of RimR2 as a positive pathway-specific regulator of rimocidin biosynthesis in Streptomyces rimosus M527
Source: Microb Cell Fact. 2023 Feb 21;22:32. doi: 10.1186/s12934-023-02039-9 (PMC9942304; doi:10.1186/s12934-023-02039-9)

**Additional file 10:**

**Figure S9.** Construction of recombinant plasmids of overexpressing *rimR*1*/rimR*2*/rimR*3*/rimR*4 gene with p*erm*E^*^ promoter.


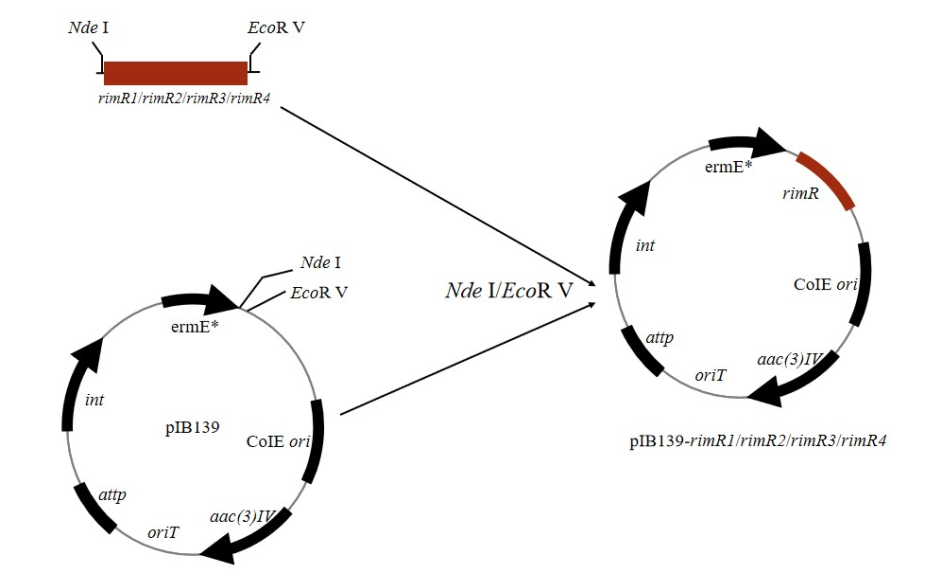

Supplement: Supplementary file 10 — Additional file 10: Figure S9. Construction of recombinant plasmids of overexpression of rimR1/rimR2/rimR3 /rimR4 gene with permE* promoter. [file 12934_2023_2039_MOESM10_ESM.docx]
