# Supplementary material for: Identification of RimR2 as a positive pathway-specific regulator of rimocidin biosynthesis in Streptomyces rimosus M527
Source: Microb Cell Fact. 2023 Feb 21;22:32. doi: 10.1186/s12934-023-02039-9 (PMC9942304; doi:10.1186/s12934-023-02039-9)

**Additional file 11:**

**Figure S10.** Detection and comparison of rimocidin production (**a**) and cell dry weight (**b**) of WT strain *S. rimosus* M527(●), recombinant strains M527-R1(■), M527-R2(▲), M527-R3(▼), and M527-R4(◆) in shake-flask culture experiment. All shake-flask fermentations were carried out in 250 ml flasks with a working volume of 40 ml at 200 rpm and 28°C. The medium was inoculated at 5% (v/v). The error bars were calculated from three different batches of fermentation.


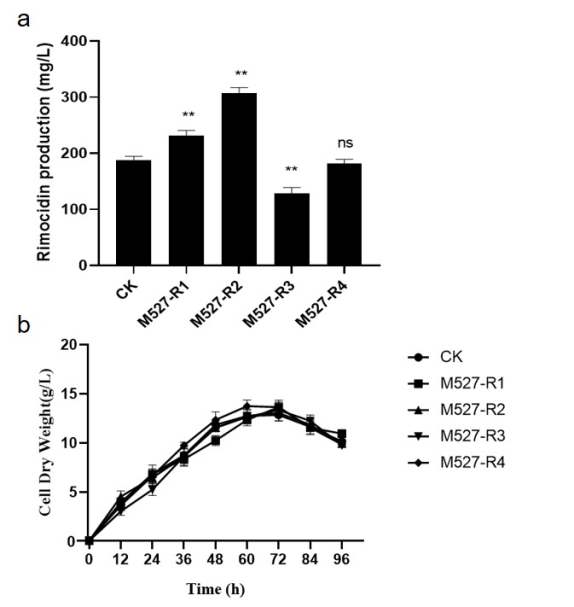

Supplement: Supplementary file 11 — Additional file 11: Figure S10. Detection and comparison of rimocidin production (a) and cell dry weight (b) of WT strain S. rimosus M527(●), recombinant strains M527-R1(■), M527-R2(▲), M527-R3(▼), and M527-R4(◆) in shake-flask culture experiment. [file 12934_2023_2039_MOESM11_ESM.docx]
