# Supplementary material for: Identification of RimR2 as a positive pathway-specific regulator of rimocidin biosynthesis in Streptomyces rimosus M527
Source: Microb Cell Fact. 2023 Feb 21;22:32. doi: 10.1186/s12934-023-02039-9 (PMC9942304; doi:10.1186/s12934-023-02039-9)

**Additional file 12:**

**Figure S11.** Detection and comparison of antifungal activities of WT strain M527, recombinant strains M527-R1, M527-R2, M527-R3, and M527-R4 against *F. oxysporum* f. sp. *cucumerinum*. Spore suspension (500 μl) of *F. oxysporum* f. sp*. cucumerinum* (1×10^6^ cfu ml^-1^) was spread and inoculated on PDA medium at 28 °C for 1 d. A agar block (4 mm in diameter) containing actively growing WT strain M527, three random recombinant strains M527-R1(**a**), M527-R2(**b**), M527-R3(**c**), and M527-R4(**d**) was aseptically placed on aforementioned PDA medium containing pathogenic fungus at 28 °C for 3-4 d. The diameter of inhibition zone was measured as antagonistic activity. Plant-pathogenic fungus *F. oxysporum* f. sp. *cucumerinum* was used as indicator strain in antifungal activities assay.


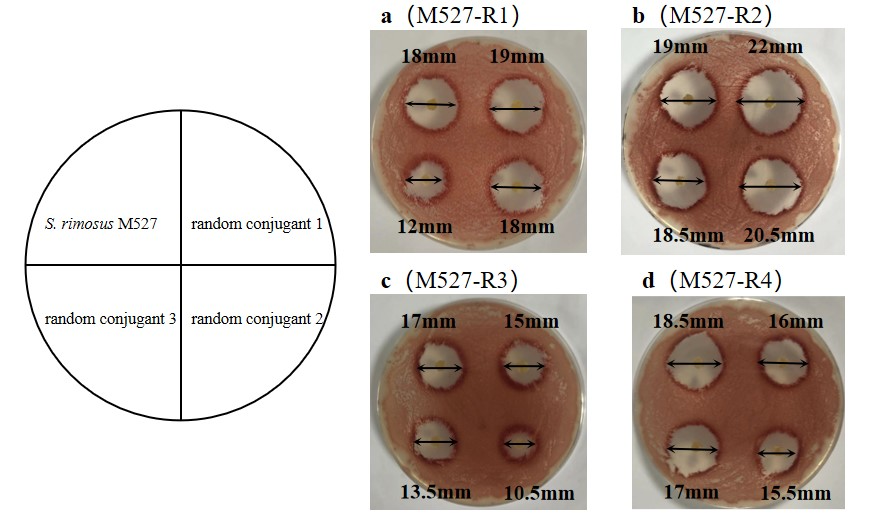

Supplement: Supplementary file 12 — Additional file 12: Figure S11. Detection and comparison of antifungal activities of WT strain M527, recombinant strains M527-R1, M527-R2, M527-R3, and M527-R4 against F. oxysporum f. sp. cucumerinum. Spore suspension (500 μl) of F. oxysporum f. sp. cucumerinum (1×10^6^ cfu ml-1) was spread and inoculated on PDA medium at 28 °C for 1 d. A agar block (4 mm in diameter) containing actively growing WT strain M527, three random recombinant strains M527-R1(a), M527-R2(b), M527-R3(c), and M527-R4(d) was aseptically placed on aforementioned PDA medium containing pathogenic fungus at 28 °C for 3-4 d. The diameter of inhibition zone was measured as antagonistic activity. Plant-pathogenic fungus F. oxysporum f. sp. cucumerinum was used as indicator strain in antifungal activities assay. [file 12934_2023_2039_MOESM12_ESM.docx]
