# Supplementary material for: Identification of RimR2 as a positive pathway-specific regulator of rimocidin biosynthesis in Streptomyces rimosus M527
Source: Microb Cell Fact. 2023 Feb 21;22:32. doi: 10.1186/s12934-023-02039-9 (PMC9942304; doi:10.1186/s12934-023-02039-9)

**Additional file 13:**

**Figure S12.** Phylogenetic tree of RimR1 and other polyene macrolide biosynthesis regulators(PAS-LuxR). ScnRII, a regulator of natamycin biosynthesis from *Streptomyces chattomnoogensis*; AURJ3M, a regulator of anreofuscin biosynthesis from *Streptomyces cureofuscus*; PimM, a regulator of natamycin biosynthesis from *Streptomyces* *natalensis*; FilF, a regulator of filipin biosynthesis from *Streptomyces filipinensis*; NysRIV, a regulator of nystatin biosynthesis from *Streptomyces noursei* A TCC 11455; PteF, a regulator of filipin biosynthesis from *Streptomyces avermitilis*; TtmRIV, a regulator of tetramycin biosynthesis in *Streptomyces ahygroscopicus*; FscRI, a regulator of candicidin biosynthesis from *Streptomyces* sp. FR-008; AmphRIV, a regulator of amphotericin biosynthesis from *Streptomyces nodosus.*


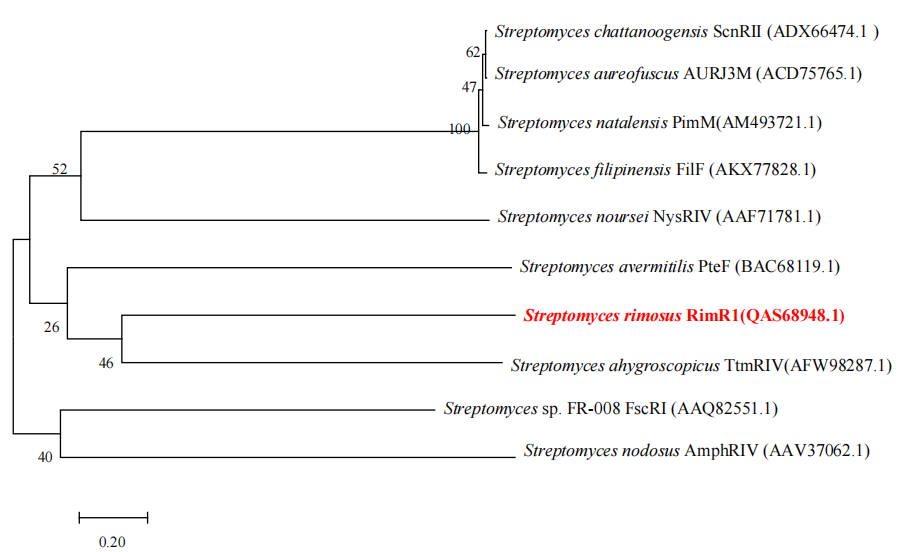

Supplement: Supplementary file 13 — Additional file 13: Figure S12. Phylogenetic tree of RimR1 and other polyene macrolide biosynthesis regulators (PAS-LuxR). [file 12934_2023_2039_MOESM13_ESM.docx]
