# Supplementary material for: Identification of RimR2 as a positive pathway-specific regulator of rimocidin biosynthesis in Streptomyces rimosus M527
Source: Microb Cell Fact. 2023 Feb 21;22:32. doi: 10.1186/s12934-023-02039-9 (PMC9942304; doi:10.1186/s12934-023-02039-9)

**Additional file 14:**

**Figure S13.** Phylogenetic tree of RimR2, RimR3, RimR4 and other polyene macrolide biosynthesis regulators (LAL). AmphRI, AmphRII, AmphRIII, AmphRVI, regulators of amphotericin biosynthesis from *Streptomyces nodosus*; TtmRI, TtmRII, TtmRIV, regulators of tetramycin biosynthesis in *Streptomyces ahygroscopicus*; FscRII, FscRIII, FscRIV regulators of candicidin biosynthesis from *Streptomyces* sp. FR-008; NysRI, NysRII, NysRIII, regulators of nystatin biosynthesis from *Streptomyces noursei* A TCC 11455.


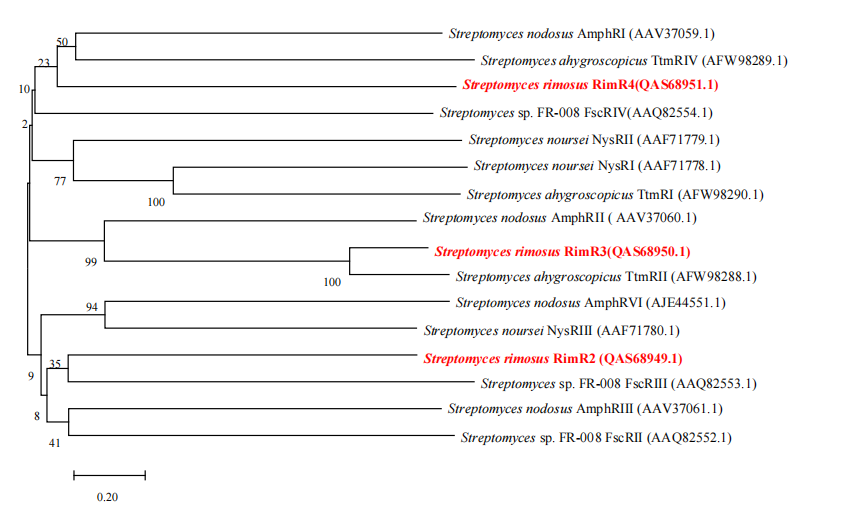

Supplement: Supplementary file 14 — Additional file 14: Figure S13. Phylogenetic tree of RimR2, RimR3, RimR4 and other polyene macrolide biosynthesis regulators (LAL). [file 12934_2023_2039_MOESM14_ESM.docx]
