# Supplementary material for: Identification of RimR2 as a positive pathway-specific regulator of rimocidin biosynthesis in Streptomyces rimosus M527
Source: Microb Cell Fact. 2023 Feb 21;22:32. doi: 10.1186/s12934-023-02039-9 (PMC9942304; doi:10.1186/s12934-023-02039-9)
Supplement: Supplementary file 15 — Additional file 15: Table S2. The primers used for deletion or expression of rimR2 gene in this study. [file 12934_2023_2039_MOESM15_ESM.docx]

**Additional file 15:**

**Table S1** The primers used for deletion or expression of *rimR*2 gene in this study

| **Primer** | **Sequence (5’-3’)** | **Source or  reference** |
| --- | --- | --- |
| P1 | CGTCGACCTGCAGGCATGCAAGCTTACCCTGATCAATGGCATCGTCGG | this work |
| P2 | CCGTACGGGGCCAGGACGGACCCGTTACGTCCCTTTCCGTCGA | this work |
| P3 | TCGACGGAAAGGGACGTAACGGGTCCGTCCTGGCCCCGTAC | this work |
| P4 | TGAGTGCTTGCGGCAGCGTGAAGCTTAGGCGGTGTCGATGGTGACCG | this work |
| P5 | ACCGTCTAGAGCGTCACCCGTACCTCCGGC (*Xba* I) | this work |
| P6 | ACCGGCGGCCGCTCACCCCACGGCGAACA (*Not* I) | this work |
| P*rimR*2-F1 | ACCGACTAGTATGCTGTATGAGCGGGAGGC (*Spe* I) | this work |
| P*rimR*2-R1 | ACCGGATATCTCACCCCACGGCGAACACCG (*Eco*R V) | this work |
| P*rimR*2-R2 | ACCGGGTACCTCACCCCACGGCGAACACCG (*Kpn* I) | this work |
| P*rimR*2-F3 | ACCGGATATCATGCTGTATGAGCGGGAGGCCGAAA (*Eco*R V) | this work |
| P*rimR*2-R3 | ACCGGGATCCTCACCCCACGGCGAACACCG (*Bam*H I) | this work |
