# Supplementary material for: Identification of RimR2 as a positive pathway-specific regulator of rimocidin biosynthesis in Streptomyces rimosus M527
Source: Microb Cell Fact. 2023 Feb 21;22:32. doi: 10.1186/s12934-023-02039-9 (PMC9942304; doi:10.1186/s12934-023-02039-9)
Supplement: Supplementary file 16 — Additional file 16: Table S3. The primers used for EMSA assay in this study. [file 12934_2023_2039_MOESM16_ESM.docx]

**Additional file 16:**

**Table S2** The primers used for EMSA assay in this study.

| **Probe** | **Sequence (5’-3’)** |
| --- | --- |
| *rimR*2-Biotin or Wild | F: TACCTACAAGAAATTGCGTATCACCTCACGCAAGGAACTCCGCGCGCGGA |
|  | R: TCCGCGCGCGGAGTTCCTTGCGTGAGGTGATACGCAATTTCTTGTAGGTA |
| *rim*R1-Biotin or Wild | F: GTCGGCCCGGGGCGTTTCATTCTCCGGATTTTCGCCACCTCGCTCGATTG |
|  | R: CAATCGAGCGAGGTGGCGAAAATCCGGAGAATGAAACGCCCCGGGCCGAC |
| *rimA*-Biotin or Wild | F: ATCACCGTGCACGAGGGCTGACGCCCGCCCGGGCAGGAGCTCGTGTCCGA |
|  | R: TCGGACACGAGCTCCTGCCCGGGCGGGCGTCAGCCCTCGTGCACGGTGAT |
| *rimH*-Biotin or Wild | F: CCTCCCGCTCCGCCCGCGGCCATGTGGCCTTCGGCTACGGCGTACACCAG |
|  | R: CTGGTGTACGCCGTAGCCGAAGGCCACATGGCCGCGGGCGGAGCGGGAGG |
| *rimG*-Biotin or Wild | F：CGCCGCGAGGAACTGCGGGAATTCCTGGCCGGGGAGTCCATCGAGACCCG |
|  | R: CGGGTCTCGATGGACTCCCCGGCCAGGAATTCCCGCAGTTCCTCGCGGCG |
| *rimF*-Biotin or Wild | F: GATCCTCGGACACCCGGCCCTGGCCCGGGCGTAACACCGCACGCACATCT |
|  | R: AGATGTGCGTGCGGTGTTACGCCCGGGCCAGGGCCGGGTGTCCGAGGATC |
| *rimD*-Biotin or Wild | F: TCTTTTGTGATCAGGATGGGGGCGCGCGGCGGCCCGGGGCCGGTGGACCA |
|  | R: TGGTCCACCGGCCCCGGGCCGCCGCGCGCCCCCATCCTGATCACAAAAGA |
| *rimC*-Biotin or Wild | F:CCCCATCCTGATCACAAAAGACTAAGGGAAATGTTAAAGGTACGGTGTGC |
|  | R:GCACACCGTACCTTTAACATTTCCCTTAGTCTTTTGTGATCAGGATGGGG |
